# maLPA1-null mice as an endophenotype of anxious depression

**DOI:** 10.1038/tp.2017.24

**Published:** 2017-04-04

**Authors:** R D Moreno-Fernández, M Pérez-Martín, E Castilla-Ortega, C Rosell del Valle, M I García-Fernández, J Chun, G Estivill-Torrús, F Rodríguez de Fonseca, L J Santín, C Pedraza

**Affiliations:** 1 Departamento de Psicobiología y Metodología de las CC, Facultad de Psicología, Instituto de Investigación Biomédica de Málaga (IBIMA), Universidad de Málaga, Málaga, Spain; 2 Departamento de Biología Celular, Genética y Fisiología, Instituto de Investigación Biomédica de Málaga (IBIMA), Universidad de Málaga, Málaga, Spain; 3 Unidad de Gestión Clínica de Salud Mental, Instituto de Investigación Biomédica de Málaga (IBIMA), Hospital Regional Universitario de Málaga, Málaga, Spain; 4 Departamento de Fisiología y Medicina Deportiva, Instituto de Investigación Biomédica de Málaga (IBIMA), Universidad de Málaga, Málaga, Spain; 5 Sanford Burnham Prebys Medical Discovery Institute, La Jolla, CA, USA; 6 Unidad de Gestión Clínica de Neurociencias, Instituto de Investigación Biomédica de Málaga (IBIMA), Hospital Regional Universitarios de Málaga, Málaga, Spain

## Abstract

Anxious depression is a prevalent disease with devastating consequences and a poor prognosis. Nevertheless, the neurobiological mechanisms underlying this mood disorder remain poorly characterized. The LPA1 receptor is one of the six characterized G protein-coupled receptors (LPA1–6) through which lysophosphatidic acid acts as an intracellular signalling molecule. The loss of this receptor induces anxiety and several behavioural and neurobiological changes that have been strongly associated with depression. In this study, we sought to investigate the involvement of the LPA1 receptor in mood. We first examined hedonic and despair-like behaviours in wild-type and maLPA1 receptor null mice. Owing to the behavioural response exhibited by the maLPA1-null mice, the panic-like reaction was assessed. In addition, c-Fos expression was evaluated as a measure of the functional activity, followed by interregional correlation matrices to establish the brain map of functional activation. maLPA1-null mice exhibited anhedonia, agitation and increased stress reactivity, behaviours that are strongly associated with the psychopathological endophenotype of depression with anxiety features. Furthermore, the functional brain maps differed between the genotypes. The maLPA1-null mice showed increased limbic-system activation, similar to that observed in depressive patients. Antidepressant treatment induced behavioural improvements and functional brain normalisation. Finally, based on validity criteria, maLPA1-null mice are proposed as an animal model of anxious depression. Here, for we believe the first time, we have identified a possible relationship between the LPA1 receptor and anxious depression, shedding light on the unknown neurobiological basis of this subtype of depression and providing an opportunity to explore new therapeutic targets for the treatment of mood disorders, especially for the anxious subtype of depression.

## Introduction

Depression diagnoses are growing at an alarming rate. Depression is currently the fourth most diagnosed disease in the world and is projected to become the most diagnosed by 2030.^1^ Suffering from depression increases the risk of asthma, diabetes, obesity, cardiovascular problems and mortality.^2^ Moreover, comorbidity between anxiety and depression in clinical and epidemiologic samples is very frequent,^3^ and nearly half of those suffering from depression are also diagnosed with an anxiety disorder^4^ or what is called ‘anxious depression'.^5^ The most clinically relevant definitions for anxious depression use either dimensional or syndromic criteria, depending on whether depression is accompanied by subthreshold anxiety symptoms^6, 7^ or at least one comorbid anxiety disorder, respectively.^7^ Despite the high prevalence and devastating impact of this subtype of depression, the underlying neurobiological mechanisms of mood disorders in general of anxious depression in particular remain poorly understood.

Identifying the biological factors that increase the risk of developing mood disorders is essential for finding new potential therapeutic targets. The LPA1 receptor may be one such example. This receptor is one of the six G protein-coupled receptors through which lysophosphatidic acid (LPA, 1-acyl-2-sn-glycerol-3-phosphate) engages multiple intracellular signalling pathways, such as the Rho, PPC, Ras and PI3K pathways (reviewed in Riaz *et al.*^8^). The anatomical distribution of LPA1 receptors has been described both in adult rodent and in human, using functional [^35^S]GTPγS autoradiography and immunohistochemical procedures, respectively. The highest LPA1 receptor density was observed in myelinated areas of white matter such as the corpus callosum and internal capsule but also in the hippocampus, frontal cortex, amygdala and striatum,^9, 10, 11, 12, 13, 14, 15^ key emotion-processing regions (reviewed in Krishnan and Nestler^16^).

It has recently been shown that the LPA1 receptor is involved in emotional regulation. Notably, mice lacking the LPA1 receptor exhibit emotional dysregulation,^17^ cognitive alterations on hippocampal-dependent tasks and dysfunctional coping in response to chronic stress.^13^ Furthermore, the lack of this receptor compromises the morphological and functional integrity of the key limbic circuit.^13, 17^ Dysfunctional changes within these highly interconnected ‘limbic' regions have been implicated in depression and in the actions of antidepressants (reviewed in Krishnan and Nestler^16^). Moreover, the absence of the LPA1 receptor alters adult neurogenesis in the hippocampus,^10, 13^ induces exaggerated endocrine responses to emotional stimuli,^17^ causes hypoactivity^18^ and impairs adaptation of the hypothalamic–pituitary–adrenal axis after chronic stress,^13^ all of which are factors that have also been strongly correlated with depression (reviewed in Boucher *et al.*^19^). In addition, the absence of the LPA1 receptor has been reported to increase anxiety-like responses.^20, 21^ Most clinical and epidemiological data suggest that the onset of anxiety usually precedes the onset of depression.^22, 23^

By contrast, a recent study using a rodent embryonic fibroblast cell line reported that several antidepressants (such as tricyclic and tetracyclic antidepressants and selective serotonin or noradrenaline reuptake inhibitors) can induce different cellular responses through the LPA1 receptor.^24^ Therefore, LPA1 receptor signalling may be involved in the actions of these drugs and thus in some of their therapeutic actions.^25^

Altogether, these data lead us to suspect that the LPA1 receptor may be involved in the pathogenesis of depression that is accompanied by anxiety.

In this context, to elucidate the involvement of the LPA1 receptor in mood, we examined in normal wild-type (wt) and in maLPA1-null mice, hedonic behaviour, nest building test to model loss of energy or decreased motivation in major depression,^26^ and the forced swimming test (FST) and the tail suspension test (TST) were used to monitor helplessness in both genotypes. Furthermore, conditioned and unconditioned fear related to generalised anxiety disorder and panic disorder, respectively, has been examined.^27, 28^ In addition, in limbic and extralimbic regions implicated in depression and the antidepressant action of drugs, c-Fos expression was assessed as a measure of the functional activity induced by behaviour (female urine sniffing test (FUST) and TST),^29^ followed by a matrix of correlation for functional brain mapping. Further, the ability of antidepressant treatment with desipramine to induce behavioural improvements and functional brain normalisation was examined. Finally, according to validity (face, construct and predictive)^30^ criteria and considering the results of this study and accumulated data with studies using animals lacking the LPA1 receptor, maLPA1-null mice are proposed as an animal model of mixed depressive-anxiety phenotype.

## Materials and methods

### Animals

The generation and characterisation of the Malaga variant of LPA1-null mice (maLPA1-null mice), derived from the original colony of Contos *et al.*,^11^ have been described in previous works.^10, 31^ For all of the experimental procedures, 3-month-old-male mice were used. Mice were housed in groups of 4 on a 12 h light/dark cycle (lights on at 0700 hours), with water and food provided ad libitum. Experiments were conducted between 0900 hours and 1500 hours. Animals were housed in groups of 4 per cage except during the saccharin consumption test and the nest building tests, for which mice were housed individually. For every behavioural experiment, an independent set of animals was used (*n*⩾6 for every experimental group), except for testing the effects of chronic antidepressant treatment on motivation and despair (*n*⩾5), which were performed in the same randomized cohort of mice.

Procedures were approved by the Ethics Committee of Malaga University (CEUMA: 2012-0006-A; 2012-0007-A) and performed in compliance with European animal research laws (European Communities Council Directives 2010/63/UE, 90/219/CEE, Regulation (EC) No. 1946/2003) and Spanish National and Regional Guidelines for Animal Experimentation and Use of Genetically Modified Organisms (Real Decreto 53/2013, Ley 32/2007, and Ley 9/2003, Real Decreto 178/2004, Decreto 320/2010).

### Behavioural testing

General conditions of all of the behavioural tests are provided in the Supplementary Information.

### Hedonic testing

#### Saccharin preference test

Animals were individually housed to assess fluid consumption for each individual mouse. After a week of habituation to the presence of the two drinking bottles, mice were submitted to a water versus saccharin two-bottle preference test (saccharin sodium salt hydrate; Sigma-Aldrich, Madrid, Spain). Saccharin preference was calculated according to the following formula: saccharin preference=(saccharin intake)/(saccharin intake+water intake) × 100, as previously described.^32^ Moreover, total liquid intake was recorded (Supplementary Figure S1). Chronic effects of antidepressant on saccharin preference were also assessed. For a more detailed description of the procedure, see the Supplementary Methods.

#### Female urine sniffing test

The FUST was administered as previously described by Malkesman *et al.*,^33^ and was used to monitor reward-seeking activity in rodents together with the saccharin preference test. Olfactory function was also assessed to rule out sensory deficits that could interfere with the results. See the Supplementary Methods and Supplementary Figure S2 for further details.

#### Nest building test

In the first experiment, nest building was assessed at 24 h intervals for three consecutive days. A second experiment was conducted one week later. A Nestlet was provided at 1000 hours, and the nest was evaluated 1 h (1100 hours) and 4 h (1400 hours) later.

The scoring system to assess nest quality was based on Deacon's standardized scale^34^ from 1 to 5. An additional study was carried out to assess the effects of an antidepressant on nesting behaviour. For a more detailed description, see the Supplementary Methods.

### Behavioural despair test

#### Forced swim test

A modified version of the FST described by Cryan *et al.*,^35^ was used. Animals were placed individually into a Plexiglas cylinder (Malaga, Spain; 30 × 10 cm) filled with 23±1 °C water to a depth of 25 cm for 6 min, and behaviour was recorded using a video camera positioned in front of the tank.

An additional study was conducted to determine the chronic effect of antidepressants in this test. See the Supplementary Methods for further details.

#### Tail suspension test

For this assessment, an automatised tail suspension test (Panlab Harvard apparatus, PanLab, Barcelona, Spain) was used.

Animals were suspended by their tails with adapted adhesive tape and attached to a hook that was coupled to a computer-assisted device for measuring movement (Panlab). Each testing session lasted 6 min, during which the performance of each mouse was evaluated. Immobility (hanging upside down without any motion to escape from the unpleasant situation), energy and power of motion (PM) were assessed as additional parameters of the quality of struggling. All of the parameters were analysed using a computerised system connected to the apparatus.

An additional study was carried out to determine the effects of antidepressants on the parameters examined in both genotypes (Supplementary Information).

### Anxiety-like test

#### Passive avoidance

The elevated T-maze (ETM), an animal model derived from the elevated plus maze, was used to assess passive avoidance.^28^ The apparatus consists of three arms in an elevated maze, two open arms and one enclosed arm of equal dimensions. The size of the test apparatus used was 50 × 12 cm. One arm, enclosed by walls 40 cm high, was perpendicular to two opposing open arms. To assess passive avoidance, the animals were placed at the distal end of the enclosed arm, facing the intersection. Latencies to leave the enclosed arm over three consecutive trials were recorded.

#### Escape

One-way escape from the open arm of the ETM was measured (representing unconditioned fear).^36^ For this purpose, latencies to leave one of the open arms over three consecutive trials were recorded.

In addition, locomotion (distance travelled (cm)) and velocity (cm s^−1^) were registered using a video tracking system (Ethovision XT, Noldus, Wageningen, The Netherlands; Supplementary Information).

### Histology

#### Immunohistochemistry

Ninety minutes after completing the FUST or TST (both naive or treated with desipramine), mice were intracardially perfused with 0.1 m phosphate-buffered saline, pH 7.4 (PBS), and 4% paraformaldehyde solution in PBS.^37^ Brains were post-fixed, immersed in 30% sucrose solution to cryoprotect the tissues, and cut into coronal sections (50 μm) while frozen, using a freezing microtome.

Sections were processed for immunohistochemistry by using the peroxidase conjugated extrAvidin method with diaminobenzidine as the chromogen, as described previously.^10, 13, 17, 38^ The primary antibody used was rabbit anti-c-Fos (SC-52, Santa Cruz Biotechnology, Santa Cruz, CA, USA; diluted 1:1000) to detect neuronal activation. The secondary antibody was biotin-conjugated swine anti-rabbit (E0353 Dako, Glostrup, Denmark; 1:500; Supplementary Information and Supplementary Figure S3).

#### Cell counting

Cell counting was conducted in the interconnected limbic and extralimbic regions that have been implicated in depression, antidepressant actions and anxiety.

The hippocampus (DG, CA1 and CA3), the medial prefrontal cortex (mPFC) (including both the infralimbic (IL) and prelimbic (PL) cortices), the central and basolateral amygdala (CeA and BLA), nucleus accumbens (NAc, both the core and shell), habenula (lateral (LHb) and medial (mHb)), the ventral tegmental area (VTA), the dorsal raphe nucleus (DRN), the paraventricular nucleus of the hypothalamus (PVN) and the dorsal and ventral periaqueductal area (dPAG and vPAG) were quantified. For this purpose, each area of interest was delineated according to the criteria set forth by Paxinos & Franklin (2001).^39^ The number of cells per unit of volume was calculated for each animal (Supplementary Material).

### Statistical analysis

For statistical analysis, extreme upper or lower values were identified as outliers in box plots and discarded from the analysis and Shapiro–Wilk test was used to assess normal distribution of data. Levene's test was used to test the assumption of homogeneity of variance.

#### Behavioural test

The duration of behaviours exhibited by animals of both genotypes during the FST and TST, the latency of the first immobility period in the FST, and the nest score assessed at 24 h intervals were analysed using Student's *t*-test. For maLPA1-null mice and their wt controls, saccharin preference at different concentrations, sniffing duration for distilled water and fresh urine from females in oestrus, nest scores assessed after 1 and 4 h, minute-by-minute FST data for every dependent variable, passive avoidance and escape in the T-maze, and the effects of treatments with antidepressants in FST and TST were analysed using repeated-measures ANOVA and *post-hoc* Fisher's least significant difference (LSD) analysis.

#### Brain functional activity connectivity analysis

First, we assessed whether there were differences between genotypes at baseline (Supplementary Figure S4a; *n*=5 per genotype). To determine the functional activity induced by behaviour (FUST and TST) (*n*=7 or 5 per genotype, respectively), the rate of change with respect to the basal activity of c-Fos expression was established for both genotypes (Supplementary Figure S4b). To examine behaviour-induced functional differences between groups, Student's *t*-test was applied to every structure examined (Supplementary Material and Supplementary Figure S4a). An additional study was carried out to determine the effect of antidepressant in the functional activity induced by TST (Supplementary Material).

To assess whether there are differences between genotypes in the brain's functional activity related to hedonic (FUST) or coping stress behaviour (in TST), correlation analyses between behaviours and regional c-Fos expression were conducted.

For brain functional mapping of each group of animals (wt and maLPA1-null mice at basal level and after behaviours), all possible pairwise correlations between the Fos signal in the 16 regions examined involved in mood regulation were determined by computing Pearson correlation coefficients. In addition, interregional correlations were further examined after TST under vehicle or desipramine treatment. Each complete set of interregional correlations was plotted in colour-coded correlation matrices using MATLAB software (MathWorks, Natick, MA, USA).

Finally, functional networks (at rest and after behaviour) of the brain regions involved in regulating mood were constructed by thresholding interregional correlations in each group of animals. This analysis used Pearson's *r* correlations ⩾0.8, uncorrected for multiple comparisons, and network graphs were generated (Supplementary Figures S4c and d).

## Results

### Behavioural tests

#### Hedonic testing

**Figure 1:**
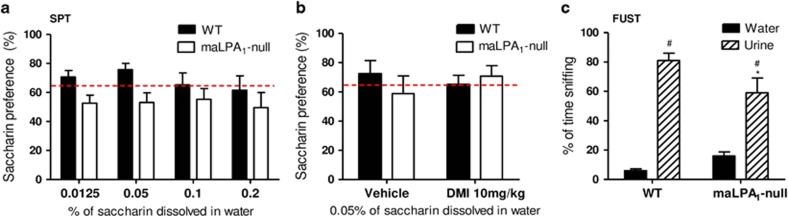
Anhedonic behaviours in mice lacking the LPA1 receptor. (**a**) In the saccharin preference test, maLPA1-null mice displayed a preference below the anhedonic threshold set at 65% in all doses, showing significant differences from the preference of wt mice (*n*=7 per genotype). (**b**) Chronic desipramine treatment leads to improvement in motivation of maLPA1-null animals reaching levels above the anhedonic threshold (**c**) In female urine sniffing tests, maLPA1-null mice exhibited a significant reduction of the percentage of time sniffing oestrogenic female urine compared to wt animals (*n*= 6 per genotype). *P<0.05 time compared with wt. ^#^*P*<0.05 time spent sniffing urine versus water. DMI, desipramine; FUST, female urine sniffing test; SPT, saccharin preference test.

Repeated-measures ANOVA revealed an effect of genotype (F(1,19)=4.86; *P*<0.05): the preference for saccharin was significantly higher in wt than in null animals (Figure 1a). However, saccharin concentration did not influence the Preference Index (F(3,57)=0.53; *P*⩾0.05). These data suggest that the lack of LPA1 receptors induced an anhedonic profile that could be reverted by antidepressant treatment (F(1,6)=2.40; *P*<0.05). Thus, after chronic treatment maLPA1-null mice displayed a preference above the anhedonic threshold set at 65% (Supplementary Materials and Figure 1b).

Measurements of social motivation can be used to evaluate hedonic behaviour. For this reason, the FUST was conducted in wt and maLPA1-null mice. A genotype × odorant interaction effect was observed (F(1,12)=4.96; *P*<0.05); *post-hoc* analysis revealed that both groups sniffed female urine significantly longer than distilled water (LSD: *P*<0.0001). However, wt mice spent significantly longer than maLPA1-null mice sniffing female urine (LSD: *P*<0.05) (Figure 1c). Thus, the lack of LPA1 receptors affected reward-seeking activity, supporting the role of the LPA/LPA1 receptor signalling pathways in hedonic responses.

### Nest building test

**Figure 2:**
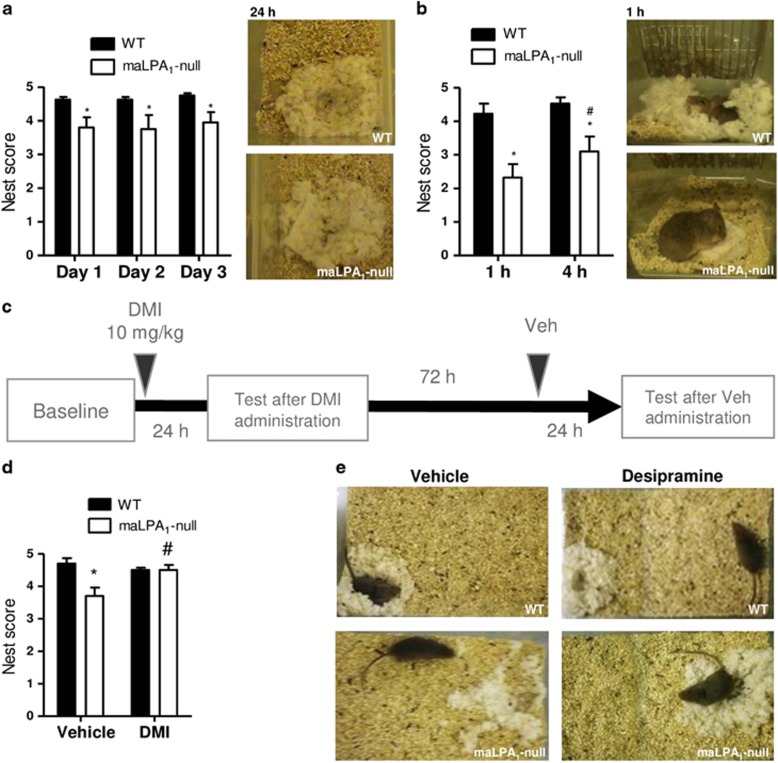
Impaired nesting behaviour in maLPA1-null mice is reverted by desipramine treatment. The quality of the nest built by each genotype was assessed after 24 h (**a**) or after 1 h and 4 h (**b**) (*n*=10 per genotype). (**c**) Experimental procedure. (**d**) Nest scores 24 h after administration of DMI or vehicle. (**e**) Representative images from the test for both genotypes. *P<0.05 **P<0.01 difference from wt; ^#^*P*<0.05 difference between time points. DMI, desipramine.

After 24 h, the wt mice had built near-perfect nests, whereas the maLPA1-null mice frequently built flat nests or left a significant amount of the Nestlet untorn (day 1: *t*_18=_−2.65; *P*<0.05; Day 2: *t*_18=_−2.07; *P*=0.052; Day 3: *t*_18=_−2.59; *P*<0.05; Figure 2a). The impaired nesting behaviour in the maLPA1-null mice was even more evident when mice were observed at 1 and 4 h after the Nestlet was introduced in the cage (difference by genotype: F(1,18)=19.16, *P*<0.001; difference by time interval: F(1,18)=5.04, *P*<0.05), considering that the wt mice shredded the whole Nestlet and built a functional nest within 1 h (Figure 2b). Nevertheless, an interesting observation is that the maLPA1-null mice were usually found sleeping on top of the Nestlet regardless of the quality of the nest built (for example, Figure 2b), suggesting that they identified the Nestlet as nesting material and were motivated to use it as a nest. Desipramine improves nest construction in maLPA1-null mice after 24 h (F(1,8)=20.51, *P*<0.005). Learning or memory effects after several tests exposure could explain some of the improvements observed, but the administration of vehicle 72 h after administrating desipramine did not affect nest building. Thus, an increment of energy and motivation induced by desipramine could at least partly explain the improvement observed in nest building in maLPA1-null mice (LSD Figure 2d).

### Behavioural despair tests

In the FST, immobility or floating behaviour has been associated with depression-like behaviour. Surprisingly, Student's *t*-test revealed that the duration of immobility was significantly reduced in the maLPA1-null mice compared with the wt mice (*t*_10_=3.24; *P*<0.05). The reduced immobility time was accompanied by an increase in climbing/struggling behaviour (*t*_10_=−3.12; *P*<0.05). No differences in swimming time were observed between the genotypes. However, compared to the wt mice, the maLPA1-null mice showed a reduced latency of the first immobility period, a more sensitive measure of depression-like behaviour than the duration of immobility^40^ (*t*_10_=3.34; *P*<0.01). This effect was reverted by chronic antidepressant administration (F(1,16)= 4.44 *P*⩽0.05). Moreover, maLPA1 null mice increased swimming behaviour (F(1,16)=6.85 *P*<0.05; LSD in Supplementary Figure S5e).

**Figure 3:**
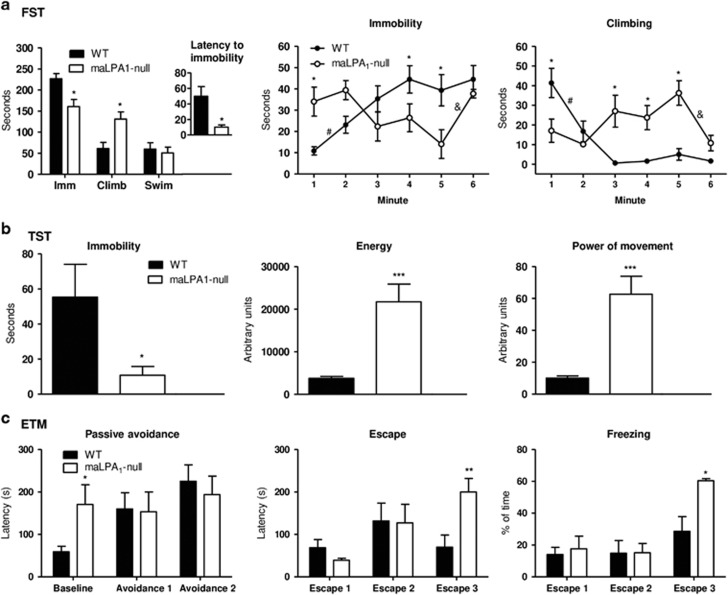
Mice lacking the LPA1 receptor showed altered stress-coping behaviour (FST and TST) together with fear and anxiety (ETM). (**a**) MaLPA1-null mice exhibited reduced immobility and increased climbing compared to wt mice. The behavioural patterns differed between the two genotypes for all tests (*n*=7 per genotype). (**b**) In TST, maLPA1-null mice exhibited lower immobility and higher energy and PM levels than the control group (*n*=8 per genotype). (**c**) Compared with the wt mice, maLPA1-null mice showed higher baseline avoidance in the ETM, indicating anxiety-like behaviour, without habituating during the test. In the third trial of the escape test, they took more time to leave the open arm, which is associated with the high proportion of freezing time (*n*=9 per genotype). *P<0.05 **P<0.01 ***P<0.001 difference from wt; ^#^*P*<0.05 or ^&^*P*<0.05 difference between time points in wt or maLPA1-null mice, respectively. ETM, elevated T-maze; FST, forced swim test; Imm, immobility; TST, tail suspension test.

A minute-by-minute behavioural analysis in the FST shows significant results for immobility (F(5,60)=5.34, *P*<0.001), swimming (F(5,60)=2.63, *P*<0.05) and climbing (F(5,60)=6.91, *P*<0.001). However, the behavioural patterns exhibited differed between the genotypes. Thus, as wt showed reduced immobility and increased climbing time at the beginning of the test and contrasting behaviour at the end, maLPA1-null mice exhibited the opposite pattern (LSD is shown in Figure 3a).

These data were corroborated by the TST. Thus, compared to wt mice, maLPA1-null mice showed reduced immobility time (*t*_14=_−2.29; *P*<0.05) and increased PM (*t*_14=_4.33; *P*<0.001) and energy (*t*_14=_=4.63; *P*<0.001) (Figure 3b).

In this sense, there is some controversy regarding the interpretation of these results (such as the increment of climbing/struggling behaviour or energy).^41, 42^ Although climbing or incremented PM and energy could be considered as active behaviour, an increase of over seven times compared with those responses without a parallel increase in swimming behaviour may be an anomalous reaction that was reduced by antidepressant treatment (Supplementary Figure S6). In fact, some authors consider that it might be a panic-like reaction to a particularly stressful situation.^41, 43^ Therefore, passive avoidance and escape behaviours were assessed.

### Anxiety-like test

Because the open arms are unpleasant, the animal will learn passive avoidance when repeatedly placed at the end of the enclosed arm and allowed to explore the maze.^28^ However, maLPA1-null mice exhibited a longer period of avoiding the enclosed arm in the first trial, with no change throughout the test, resulting in overall differences between the genotypes (F(2,30)=3.79; *P*<0.05) (Figure 3c). Working memory problems observed in null mice^21^ could explain some of the deficits observed, but at least in the first trial, memory problems cannot be the mechanism responsible for the observed deficits.

Regarding escape behaviour, in contrast to what happens in the enclosed arm, the latency to leave the open arm usually does not change with successive trials.^28^ Nevertheless, maLPA1-null mice showed increased time to escape from the open arm over the course of the test (F(2,30)=4.69; LSD: *P*<0.05 trial 3 versus 2 and trial 2 versus 1), and in the third trial, they showed a significantly higher latency to leaving the aversive arm than did wt mice (LSD: *P*<0.01). An ethological analysis of behaviour revealed that, although the lack of LPA1 receptor did not affect the motor behaviour (Supplementary Figure S7), increased escape latency was accompanied by increased freezing (*t*_12=_3.03; *P*<0.01) (Figure 3c), a measure of fear.^17, 44^ This behaviour is reminiscent of the sensitization that occurs over time towards fear responses to the test after repeated exposure, and it excludes the possibility that a deficit in learning and working memory could explain this behaviour.

### Cell counting and functional connectivity

**Figure 4:**
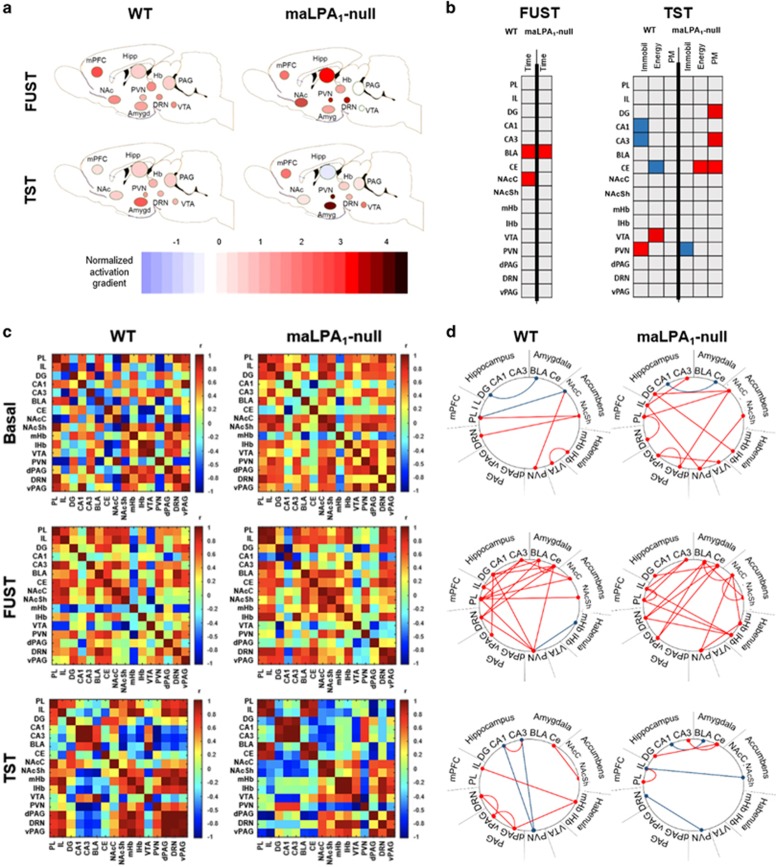
Functional connectivity in wt and maLPA1-null mice. (**a**) Activity of the limbic and extralimbic regions involved in mood after behaviour was normalised to the relevant basal group. Structures were grouped into major brain subdivisions. (**b**) Significant correlations between c-Fos activation and behavioural parameters in the FUST and TST. (**c**, **d**) Data are presented for the three conditions: basal (upper), after FUST (middle) and after TST (lower). (**c**) Interregional correlation matrices reveal different limbic maps in the absence of LPA1 receptor, especially in basal and TST conditions. (**d**) Strongly correlated levels of c-Fos among structures (*r*⩾±0.8; *P*⩽0.05) are shown in the diagrams. Red lines indicate positive correlation, while blue lines illustrate negative correlations. BLA, basolateral amygdala; CA1, cornus ammonis 1; CA3, cornus ammonis 3; CE, central amygdala; d/vPAG, dorsal/ventral periaqueductal grey matter; DG, dentate gyrus; DRN, dorsal raphe nucleus; FUST, female urine sniffing test; IL, infralimbic cortex; lHb, lateral habenula; mHb, medial habenula; mPFC, medial prefrontal cortex; NAc, nucleus accumbens; NAcC, nucleus accumbens core; NAcSh, nucleus accumbens shell; PL, prelimbic cortex; PVN, paraventricular nucleus; TST, tail suspension test; VTA, ventral tegmental area.

To establish the change with respect to baseline in the activation of structures related to mood and hedonic behaviour, data were normalised to baseline levels (as shown in Figure 4a). After the FUST, maLPA1-null mice showed increased functional activation, compared with baseline, in the PVN, DRN, NAc and hippocampus. In the TST, the clearest changes in maLPA1-null mice were observed in the BLA, DRN and mPFC. Independently, the estimation of the numerical density of c-Fos positive cells (c-Fos per mm^3^; Supplementary Figure S4a) revealed that the number of c-Fos+, that is, activated, cells in the NAc core (*t*_11_ =−2.85; *P*<0.05) and shell (*t*_11_=−2.94; *P*<0.05), LHb (*t*_11_ =−2.82; *P*<0.05) and dorsal raphe nucleus (*t*_10_ =−2.50; *P*<0.05) was greater in maLPA1-null mice than in wt mice. Regarding the TST, maLPA1-null mice displayed higher c-Fos expression than wt mice in the IL (*t*_6_=−2.76; *P*<0.05), BLA (*t*_6_=−2.58; *P*<0.05), CeA (*t*_6_ =−3.77; *P*<0.01) and PVN (*t*_6_=−3.66; *P*<0.01). Treatment of null mice with antidepressants normalised the activity of BLA (F(1,11)=2.16 *P*=0.10) and PVN (F(1,11)=7.25 *P*<0.05) regions after TST, two brain areas involved in stress-coping behaviour (LSD in Supplementary Figure S8).

Next, the correlation between regional c-Fos expression and behaviour was investigated. Only strong relationships (Pearson's *r*⩾0.8, *P*<0.005) were considered. The sign of the correlation coefficient and the correlation between the regional activation and behaviour are indicated in Figure 4b and Supplementary Figure S4c.

Because depression is associated with an abnormal topological organisation of brain networks, including regional connectivity (reviewed in refs 43,44), the neural network organisation in both genotypes was explored. For this purpose, we computed a complete set of interregional correlations in the groups of mice both at baseline and after completing behavioural tests (Figure 4c). This analysis allowed us to identify sets of brain regions in which c-Fos expression co-varied across mice, presumably as components of a network that are co-active during the behaviours. As shown in Figure 4d, network graphs for each condition were subsequently generated by considering only the strongest significant correlations (Pearson's *r*⩾0.8, *P*<0.005). The patterns of connectivity between brain regions differed as a function of genotype both at baseline and after behavioural tests. As revealed by the data, regional immediate gene expression has a genotype-dependent, patterned relationship with hedonic and stress-coping behaviour (Figures 4c and Supplementary Figures S4b–d).

Notably, the identification of sets of activated regions acting as ‘networks' does not necessarily mean that the regions are directly connected from a neuroanatomical perspective.

## Discussion

The identification of biological factors that increase the risk of developing mood disorders is useful for advancing effective prevention strategies and for finding new potential therapeutic targets. In particular, revealing the unknown neurobiological bases of the mixed depressive-anxiety phenotype is crucial for improving diagnosis, prognosis and treatment. The results of our study emphasise that the LPA1 receptor is essential for mood, showing that the downregulation of this receptor may be involved in inducing primary symptoms of depression accompanied by anxiety and in affecting the reward and emotional regulation brain circuits that normally serve to guide attention towards the consumption of natural rewards and to regulate responses to aversive experiences.

Anhedonia, which can broadly be defined as a diminished capacity to experience pleasure, is a core symptom in mood disorders.^45, 46^ Because a preference for saccharin at <65% of the total drinking liquid^32^ is an indicator of anhedonia, it is possible that the null animals exhibited a decreased ability to experience pleasure. The antidepressant treatment increased the motivation to choose the pleasurable solution. In addition, considering that social communication in rodents occurs primarily through olfactory cues^33^ and that a decrease in sexual interest is observed in depressed patients,^47^ the time that male rodents spent sniffing oestrogenic females' urine was also used to assess reward-seeking behaviour. Consistent with the decreased preference for saccharin that occurred in the null animals, the reduction of spontaneous sniffing responses to urinary pheromones of the opposite sex exhibited by the maLPA1-null mice may indicate anhedonia and an impairment of appetitive behaviours in the absence of LPA1 receptors.

Reduced nest building has been suggested to model aspects of major depression, such as loss of energy and reduced motivation. The wt mice were able to construct a nearly perfect nest within 1 h, whereas the maLPA1-null mice showed marked impairments at 1 h and 4 h after starting the nest building. The deficits persisted at 24 h after the tests, although to a lesser extent than that observed at short time intervals, suggesting a loss of energy in this genotype. Decreased energy or increased fatigue, in addition to a loss of interest, are core symptoms of depression (ICD-10 and DSM-5);^48, 49^ therefore, the behaviours exhibited by the maLPA1-null mice are comparable to symptoms of depression, and these behaviours were rescued following treatment with an antidepressant.

Behavioural despair is characteristic of depressive disorder and can be assessed in animals using the FST and TST.^26, 50^ Although many behavioural reactions in the TST and the FST can be considered to reflect active defence mechanisms in response to an aversive environment,^51^ classically, in these tests, immobility has been interpreted as reflecting depression-like behaviour in rodents.^26, 42^ Surprisingly, animals lacking the LPA1 receptor showed not only a reduced immobility time but also increased climbing behaviour. Thus, although wt mice showed increased duration of immobility throughout the FST and reduced time spent climbing, presenting the normal reaction to this test,^42, 52^ the maLPA1-null mice exhibited the opposite response. In water, swimming may be a proper behaviour performed by an animal to avoid drowning,^42, 43, 53^ but in maLPA1-null mice, the reduction of immobile time did not lead to a concomitant increase in swimming. This pattern of behaviour can be interpreted as an anomalous reaction. A similar profile was observed during the TST, showing that compared to the wt mice, the maLPA1 mice had high values of energy and power of movement as well as reduced immobility. The behavioural pattern exhibited by mice lacking the LPA1 receptor may indicate maladjusted reactions when faced with an adverse situation. Thus, although immobility in the FST can be considered an adaptive cognitive process to an inescapable situation,^54^ increased climbing or power of movement in despair tests may be a maladaptive behaviour that compromises (endangers) the survival of the animal through exhaustion. Reduced latency to the first immobility period (interpreted as a reduction in intrinsic motivation to escape the situation),^55^ limited immobility time and increased climbing in the FST and TST have also been observed in another animal model of depression-like episodes.^56^ Owing to the validity of these tests for measuring the antidepressant effects of drugs but not depression itself, and considering that agitation could be a symptom of depression,^57^ the anomalous behavioural pattern exhibited by null animals during the despair tests may suggest depression-like behaviour. Antidepressant treatment increased the latency to the first immobility period in FST and reduced high values of energy and power of movement during TST. In this particular stressful situation, the energetic bursts of activity exhibited by maLPA1-null mice can be interpreted as excessive emotional responses^51^ similar to panic.^42, 53^

In fact, maLPA1-null mice showed abnormal emotional responsivity in the T-maze. On the basis of the assumption that unconditioned fear is associated with panic disorder, escape behaviour on the ETM has been used to assess excessive emotional reactions in animals.^28^ A careful analysis revealed that, in maLPA1-null mice, after repeated exposure, increased escape latency was accompanied by increased freezing, a behaviour that interferes with escape. Moreover, longer response latencies were observed in this genotype when first exposed to the passive avoidance platform and showed no changes throughout the test, possibly indicating an unconditioned fear response, increased basal anxiety and a habituation problem. In the maLPA1-null mice, a general hyperactivity in response to aversive stimulation may underlie the behaviour in both tests, demonstrating an anxiogenic phenotype.

Anhedonia together with increased stress reactivity is an important candidate for a psychopathological endophenotype of major depression.^45^ The alteration of LPA1 receptor could be a susceptibility factor for the presence of comorbid depression and anxiety.

Considering that dysfunctional changes within the highly interconnected ‘limbic' regions have been implicated in depression,^16^ it has been investigated whether the absence of the LPA1 receptor induced a different pattern of activation in the neurocircuit involved in mood regulation at rest. For this reason, c-Fos expression was assessed as a measure of functional activity (Supplementary Figure S3). As expected, increased c-Fos expression in CeA and CA3 was observed in maLPA1-null mice compared with control mice. Excessive limbic activation, specifically amygdala hyperactivation, may affect the ability to regulate emotion,^17^ with a negative mood bias and maladaptive processing of emotional stimuli, resulting in long-term depressive dysfunction and symptoms of anxiety.^58^

Because functional brain activity is likely relevant to the behavioural outcome, the correlation between c-Fos expression and behavioural performance of the animals was assessed. During the FUST, only in wt mice was hedonic behaviour correlated with activation of the BLA and NAc, two regions that have essential roles in regulating emotional reactivity to hedonic stimuli. No correlation between NAc and hedonic behaviour was observed in maLPA1-null mice (Figure 4b). Reduced response of NAc, along with activation of the DRN and LHb (Supplementary Figure S4a), two regions essential in mediating aversion,^59^ have been associated with a failure to trigger reinforcement mechanisms.^58^ Functional brain activity can thus explain the significant reduction in sexual interest observed in maLPA1-null mice.

Regarding TST, in wt mice, immobility was linked to functional activation of PVN and decreased activation in the hippocampus, while high energy was correlated with increased activation of the VTA and decreased activity in the CeA. Increased VTA^60^ and reduced amygdala^61^ activation have been considered to be mechanisms involved in counteracting the negative effects of stress, inducing anxiolysis and reducing depressive behaviours. By contrast, the maLPA1-null mice showed a prominent increase in excitation in the PVN that correlated negatively with the time that the animals spent immobile. However, the time of energy and PM exhibited by the animals were linked to functional activation of the CeA, together with the hippocampus in PM (Figure 4b). Hyperactivity of the amygdala and PVN, which is associated with stress-induced neuroendocrine and molecular responses of the hypothalamic–pituitary–adrenal axis, can affect the ability to regulate emotion^17^ and induce a negative mood bias to result in long-term depressive and anxious symptoms. In fact, previous data from our group revealed an increase in amygdala reactivity, which may be related to exaggerated corticosterone release after acute stress.^17^ Moreover, similar to animals with comorbid anxious-anhedonic phenotype^62^ or treated with anxiogenic drugs,^63^ maLPA1-null mice showed increased IL activity. This pattern of functional activation after the TST supports the idea that reduced immobility time and increased climbing behaviour observed in maLPA1-null mice can be interpreted as anomalous emotional responses to a stressful situation. Although the molecular mechanism responsible for the altered c-Fos expression in null mice remains unclear, the lack of LPA1 may initiate dysregulation of glutamatergic and GABAergic signalling, resulting in an imbalance between excitatory and inhibitory neurotransmission, as revealed in previous studies.^17, 64, 65^ This would at least partly explain the pattern of activation observed in maLPA1-null mice. The antidepressant treatment restored the brain activity of null mice to normal, showing a pattern similar to wt mice, and could at least partly explain the improvement in TST.

However, the brain is structurally and functionally organised into a complex network that facilitates an efficient integration and segregation of information processing and behaviour regulation. Along with functional changes in the brain, depression has been associated with disrupted brain networks (reviewed in Gong and He^66^). Altered in brain connectivity, detectable during rest, has even been used as a biomarker of depression.^66^ Strong differences in the functional connectivity patterns were detected between genotypes both at rest and after behavioural tests (Figures 4a–d). Thus, in maLPA1-null mice, unlike wt mice, after FUST, the activity of the PFC and NAc, two regions highly interconnected during reward-seeking behaviours,^46^ was desynchronized. Moreover, inhibitory effects of the hippocampus in the PVN, as an essential neural mechanism for controlling stress and regulating emotion, were not observed in maLPA1-null mice after completing the TST. Considering the data regarding functional brain activation and its link with behavioural outcomes, it is possible to conclude that these genotypes produced different functional reorganisations of the brain network.^44^

Finally, although it is not feasible to fully model depression in animals because of the heterogeneity of the syndrome with substantial comorbidity and the impossibility of modelling key symptoms of human depression, such as guilt, suicidality and sadness, animal models are essential for understanding the neurobiology of psychiatric disorders.^30^ The results presented here, along with accumulated data using LPA1 receptor knockout animals, make it possible to consider maLPA1-null mice as a valid animal model of the mixed depressive-anxiety phenotype. In fact, as further illustrated in Supplementary Table S1, this genotype meets criteria for validation as an animal model of the mixed depressive-anxiety phenotype and may provide valuable information relevant to the diagnosis and advancement of treatment for one of the most daunting areas of psychiatric disorders, anxious depression.

In summary, the LPA1-null animals may have an anxiety/depression-related phenotype, reflecting one clinically important aspect of neuropsychiatry, comorbidity. At the neural level, the absence of the LPA1 receptor impaired the functional brain map required for normal hedonic behaviour^67^ or stress coping^68^ and therefore may partially account for the maladaptive behaviours observed in this genotype. In humans, polymorphism in the LPA1 receptor has been related to augmented risk of essential hypertension. Given that stress, the main environmental cause of depression, increases the susceptibility of patients with risk alleles,^69^ genetic variants in this receptor may participate in the aetiology of depression. Here, we have identified for we believe the first time the possible relationship of the LPA1 receptor with a mixed depressive-anxiety phenotype, shedding light on the unknown neurobiological basis of this subtype of depression^5^ and generating the opportunity to explore a new therapeutic target for the treatment of mood disorders, namely the anxious-depression subtype. Thus, drugs whose therapeutic target would be the signalling pathways of LPA1 receptors may be useful for modulating mood and anxiety. In this context, the use of LPA1 receptor agonists may emerge as a new family of antidepressants with an anxiolytic profile.
