# Supplementary material for: maLPA1-null mice as an endophenotype of anxious depression
Source: Transl Psychiatry. 2017 Apr 4;7(4):e1077–. doi: 10.1038/tp.2017.24 (PMC5416683; doi:10.1038/tp.2017.24)
Supplement: Supplementary Information [file tp201724x1.doc]

***maLPA_1_-null mice as an endophenotype of anxious depression.***

Moreno-Fernández R; Pérez-Martín M; Castilla-Ortega E; Rosell del Valle C; García-Fernández MI; Chun J; Rodríguez de Fonseca R; Estivill-Torrús G; Santín LJ, Pedraza C*

* Corresponding author

Supplementary information includes:

1.- Supplementary Methods

2.- Supplementary Results

3.- Supplementary Figures

4.- Supplementary References

5.- Supplementary Table 1.

**1.- Supplementary Methods**

***Behavioural test***

Before starting the behavioural test, animals were habituated to the behavioural room for 30 minutes. The test was conducted in a testing room illuminated at 15 lux.

***Hedonic test***

*Saccharin preference test*

Saccharin was used in order to avoid a caloric impact of the sweetened liquid consumption. Saccharin solutions at increasing concentrations were used (0.0125, 0.05, 0.01 and 0.2%). Each concentration was presented in consecutive 2-day blocks. The solutions were available 23 hours/day, and the bottles were weighed and refilled during the remaining hour. The position of the bottles was varied and counter-balanced across the left and right sides of the feeding compartment (to control for the preference of some mice for a particular side).

*FUST*

Because an olfactory deficit could have a negative effect on the FUST performance, the effects of the lack of LPA_1_ receptor on olfactory discrimination were examined (Figure S2).

For this purpose, animals were housed individually. The odorant was presented in their home cage. To prepare the odour stimulus, glass pipette a small amount (10 μL) of solution was used. Two different odours were utilized (i.e., vanilla and cinnamon). At 30 minutes after the first odorant presentation, the same odorant was presented again to assess olfactory memory. As shown in Figure S2, there were no differences between genotypes, and null mice did not show olfactory memory deficits, indicating that they could accurately distinguish between odorants. Moreover, null mice did not present olfactory discrimination problems^1^ at least at 30 minutes after the presentation of the first odorant.

We also conducted a series of preliminary experiments to determine whether oestrous female urine would have a different effect on reward-seeking behaviour depending on the genotype of the animals. For this reason, we used the urine of the same genotype as the test animal.

The FUST, previously described by Malkesman et al. (2010), is divided into three phases: 1) one exposure (3 min) to a cotton swab dipped in distilled water; 2) an intertrial interval of 45 min, during which any stimulus was presented; and 3) one exposure (3 min) to a cotton swab infused with fresh urine collected from oestrous female mice of the same genotype. The sniffing duration was measured during phase 1 (distilled water) and phase 3 (female urine). All measurements of sniffing behaviour in the FUST were conducted by two independent observers blind to the genotype of the animals. Inter-rater reliability for scoring the behaviours ranged from *r* .956 to *r* 1.00 (*p* .01).

***Evaluation of nest building***

Mice were individually housed in a cage with wood chip bedding. After one week of acclimation, they were provided with a Nestlet (a 5 cm square of compressed cotton-like material weighing approximately 2.4 gr; purchased from Ancare, USA) which was placed in the cage at 10:00 a.m. Twenty-four hours later, the quality of the nest built was evaluated, and the nesting material was carefully removed and replaced with a new Nestlet for another assessment of nest building.

The scoring system for assessing nest quality was based on Deacon’s standardized scale (Deacon 2006; 2012): 1 = the Nestlet is mostly untouched (<10% shredded); 2 = part of the Nestlet is torn, but most of it remains intact (10% - 50% shredded); 3 = most of the Nestlet is torn, but more than 10% remains intact (50% - 90% shredded), or the material is scattered in the cage without forming an identifiable nest site. If the Nestlet was completely torn (> 90% shredded) and gathered into a nest, the nest was conceptually divided into four equally sized quadrants. Each nest quadrant was then given a ‘4’ score when its wall was mostly flat or a ‘5’ score when its wall was high (i.e., mostly covering the mouse’s body height when the animal is curled up on its side), and the scores of all four quadrants were averaged. Thus, scores from 4 to 5 ranged from a functional but completely flat nest (= 4) to the ‘perfect’ crater-like nest with walls that covered the mouse all around its circumference (= 5). The scoring was conducted by a trained blind to the genotype of the animals.

**Evaluation of forced swimming test (FST)**

At the start of each test, the animal was held by its tail and was gently and slowly placed into the middle of the container. The entire FST session was videotaped for later analysis.

The parameters floating (immobility, making only those movements necessary to keep its head above the water), swimming (defined as when large and horizontal movements of the forepaws were performed, leading to displacement of the body around the cylinder) and struggling/climbing (vigorous attempts to escape, defined as when the mouse showed vigorous vertical movements of the forepaws, directed against the wall of the tank, leading to displacement of the body around the cylinder) ^4,5,8^ were scored throughout the 6 min test period by two trained observers blind to the genotype of the animals and were recorded using Ethovision XT software (Noldus, Wageningen, The Netherlands). Inter-rater reliability was calculated using Pearson's correlation coefficients.

Latency to first immobility period was scored as the time between introduction of a mouse into the pool and the first moment of complete immobility of entire animal’s body for at least 2 seconds.

After finishing the test, each animal was removed from the tank and dried with a towel before being returned to its home cage.

***Elevated T-maze (ETM)***

In this test, passive avoidance was evaluated first, followed by assessment of escape behaviour.

For passive avoidance, animals were placed at the distal end of the enclosed arm facing the intersection of the arms. The time until the mouse left the arm with all four paws was recorded (baseline). The same measurement was repeated twice at 30-second intervals (avoidance 1 and avoidance 2). Escape behaviour was assessed following avoidance training (30 seconds later): animals were placed at the end of the open arm, and the time until the mouse left the arm with all four paws was recorded (escape 1). The same measurement was repeated after 30 s (escape 2). During the inter-trial intervals, the animals were placed in a Plexiglas cage. Additionally, freezing behaviour exhibited by animals during escape test was assessment by a trained observer blind to the genotype of the animals and was recorded using Ethovision XT software (Noldus, Wageningen, The Netherlands).

***Evaluation of desipramine administration in saccharin preference test, nest building test, FST and TST.***

To assess the effect of antidepressant administration on depressive behaviour, pilot studies were initially carried out. Because several studies have determined that the mouse strain used in our study responds better to antidepressants with a noradrenergic profile instead of a serotoninergic profile,^6,7^ and considering the preliminary results of pilot studies, desipramine (a selective noradrenaline (NA) reuptake inhibitor) was tested further.

For this purpose, 30 minutes before starting the TST, vehicle or antidepressant was administered to mice of each genotype. Animals were suspended by their tails with adapted adhesive tape and were attached to a hook that was coupled to a computer-assisted movement measurement device (Panlab, Spain). Times of immobility, energy and PM were recorded. Because an automatized Tail Suspension Test was used, the investigator was no blinded to the group allocation during the experiment.

To test the effects of desipramine, nesting behaviour was tested 24 h after drug administration.^8^ To exclude the possibility that the improved nest building could be due to a learning process, the animals were submitted to the experimental procedure depicted in Figure 2. Thus, animals were assessed after 24 hours of vehicle administration.

Finally, because symptomatic improvement in humans usually occurs 7-14 days after starting the treatment, and in order to demonstrate the predictive validity of the model, chronic treatment with desipramine (14 administrations) was conducted. For this purpose, animals received 2 daily administrations of desipramine or vehicle for 7 days (see Figure S5a). Then, we assessed whether the treatment was effective at ameliorating anhedonic behaviour or normalizing the behaviour exhibited by animals in a despair test (FST).

For data analysis, a factorial ANOVA (genotype x treatment) was used.

**c-Fos quantification**

The quantification was performed using a 40x lens on an Olympus BX 51 microscope, equipped with a JVC (TK-C1480E) camera and a computer-assisted stereological toolbox, using the CAST-Grid software package (Olympus, Glostrup, Denmark). The number of cells per unit of volume was calculated for each animal as the number of c-Fos immunoreactive (IR) nuclei per mm^3^ using the following formula: Nv = ∑Q−d/∑Vdis, where Nv = numerical density; ∑Q−d = total number of c-Fos IR nuclei counted; and ∑Vdis = total number of dissectors applied multiplied by Vdis. Vdis = Sd × Hd, where Sd = area of the dissector grid (counting frame) and Hd = depth of the dissector.

***Normalization of c-Fos expression data***

After behavioural tests, c-Fos expression in limbic and extralimbic regions was normalized to the appropriate basal group (i.e., c-Fos count - basal mean count)/overall s.d.) according to Ritov, Boltyansky and Richter-Levin (2015).

Baseline was calculated by quantifying the same structures as in experimental animals. For this purpose, control animals did not receive any treatment and therefore remained in their home cages until intracardial perfusion.

***Evaluation of desipramine administration in brain activation induced by coping stress behaviour (in TST).***

For evaluating the effects of desipramine in brain activation induced by TST the hippocampus (DG, CA1 and CA3), the medial prefrontal cortex (mPFC) (including both the infralimbic (IL) and prelimbic (PL) cortices), the central and basolateral amygdala (CeA and BLA), nucleus accumbens (NAc, both the core and shell), the paraventricular nucleus of the hypothalamus (PVN) and the dorsal and ventral periaqueductal area (dPAG and vPAG) were quantified. For this purpose, each area of interest was delineated according to the criteria set forth by Paxinos & Franklin, (2001).

**2.- Supplementary Results**

**Desipramine reverted depressive symptoms and reduced agitation in null mice.**

        In TST, an effect of desipramine was observed for immobility time (F(1,11)=6.72; P<0.05), with animals spending less time without showing any movement, although only wt animals exhibited a significant difference (LSD in supplementary Figure 6). Regarding energy and power of movement, the interaction genotype x treatment was significant (F(1,11)=7.51; P<0.05 and F(1,11)=8.08; P<0.05) for energy and PM, respectively). Post hoc analysis revealed that the treatment was only significant in null mice (P<0.05). Considering that agitation can be a symptom of depression, the pharmacological treatment reduced the agitation in null mice (see Figure S6).

***2.- Supplementary Figures***

**
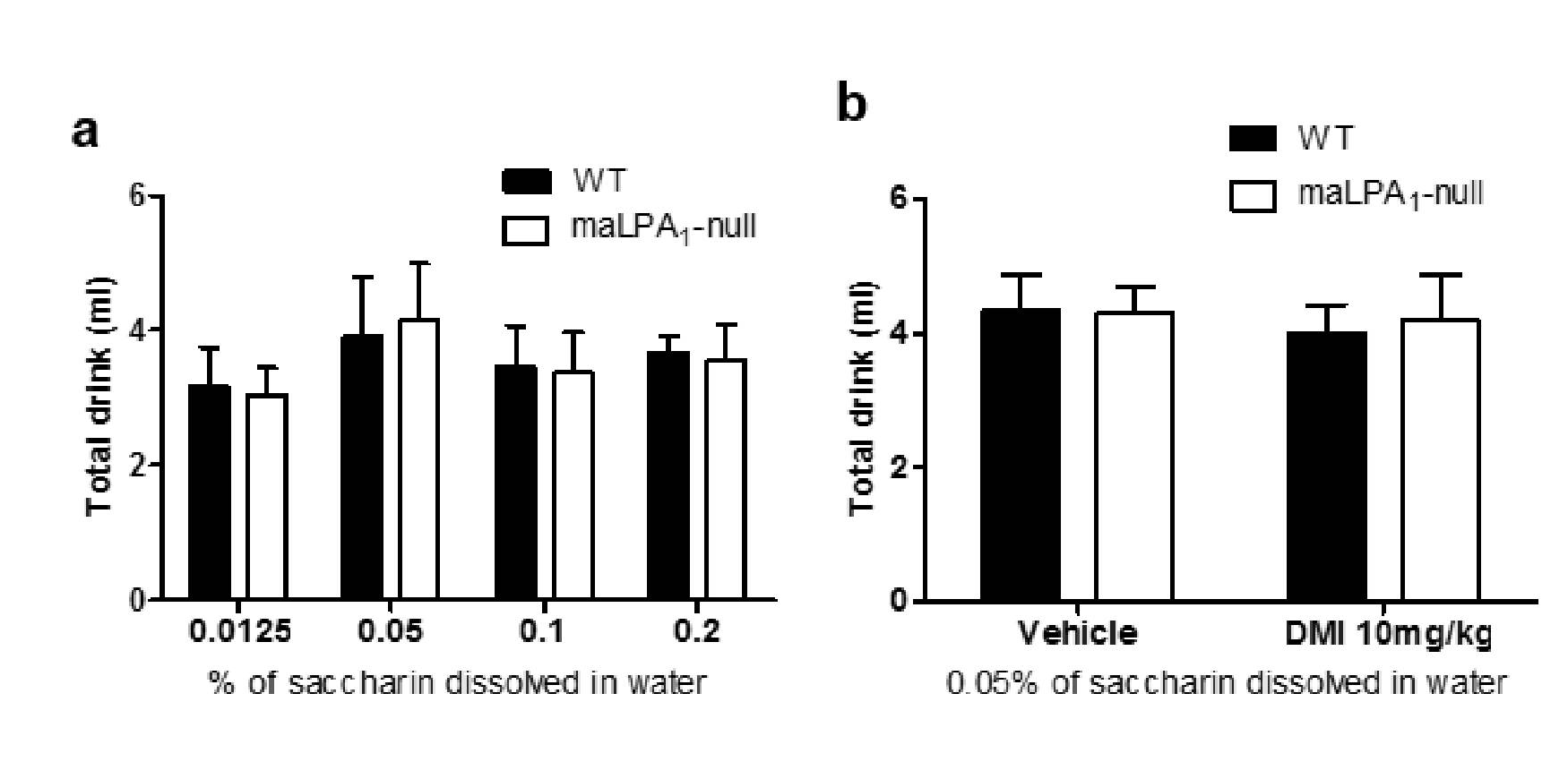
**

**Figure S1. Total liquid intake (in ml).** There were no significant differences between genotypes (**a**) nor treatment (**b**) in the total liquid intake at any concentration of saccharin.

***
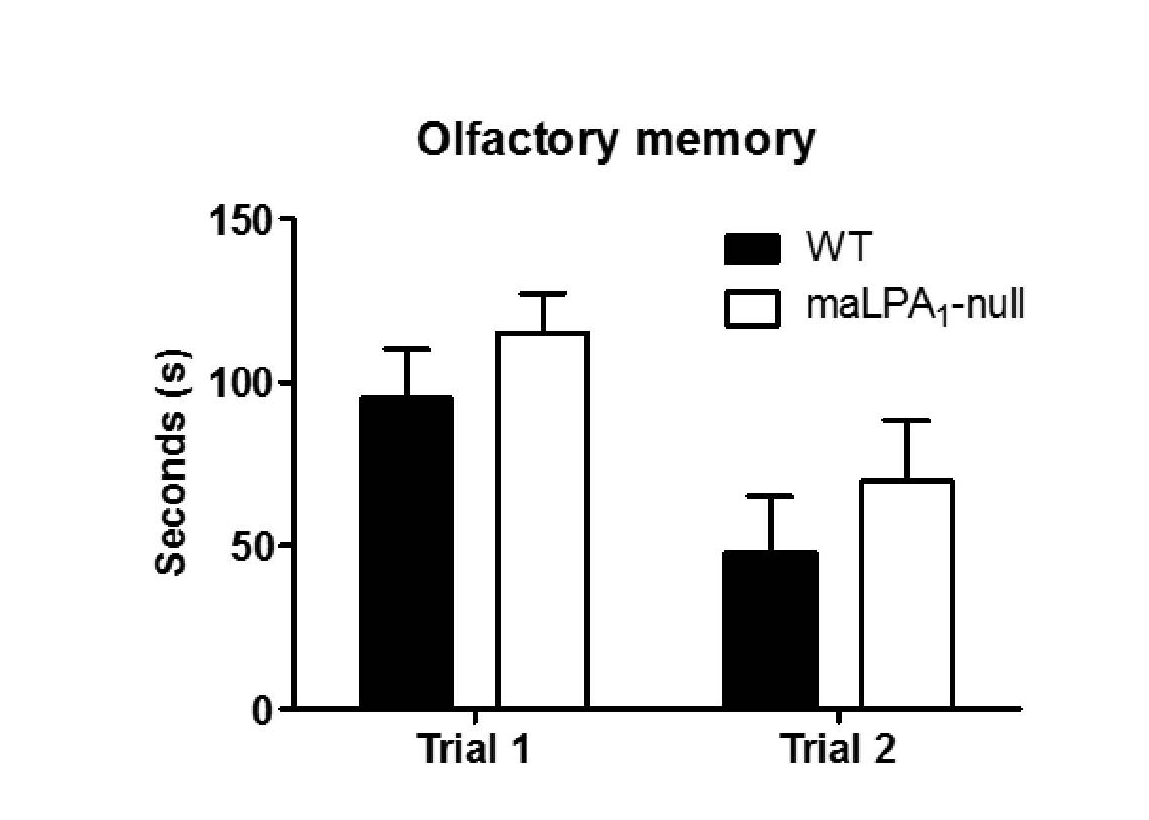
***

**Figure S2. Olfactory short-term memory is unaltered in the maLPA_1_-null animals**. Null mice did not show differences from wt animals in olfactory memory and thus were able to reduce the time of sniffing during the second presentation of an odorant presented 30 minutes before.


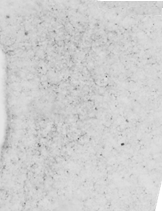

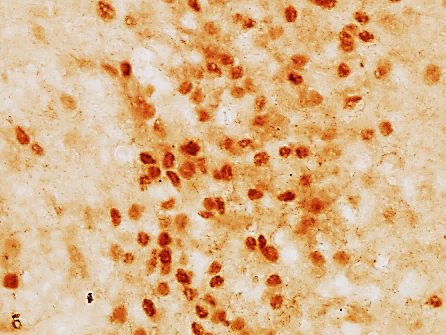

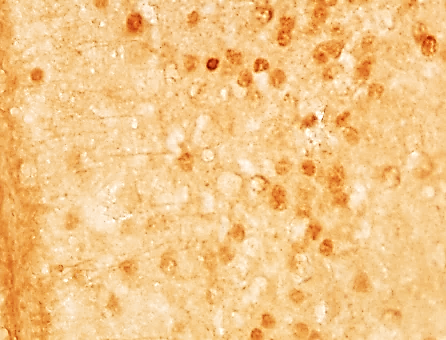


**50 µm**


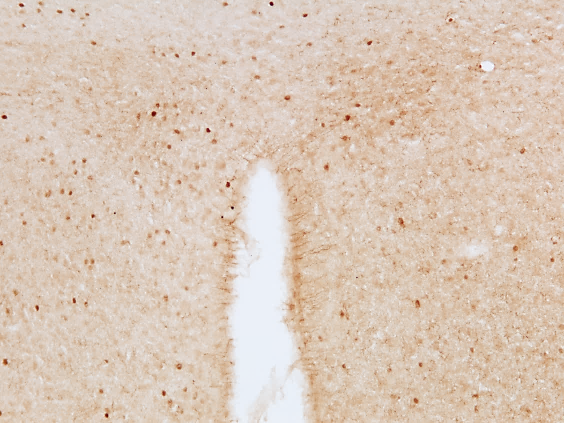

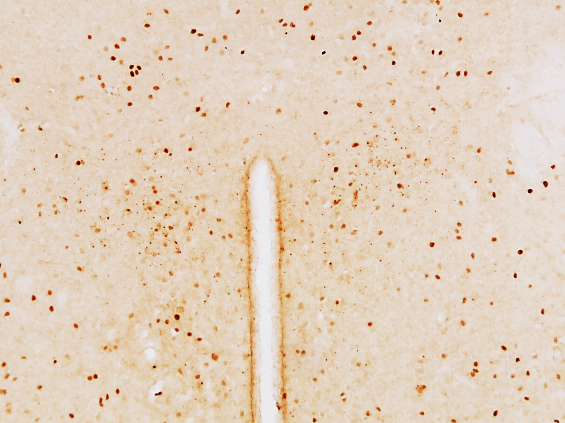

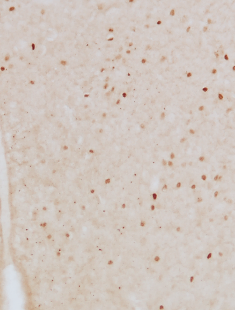

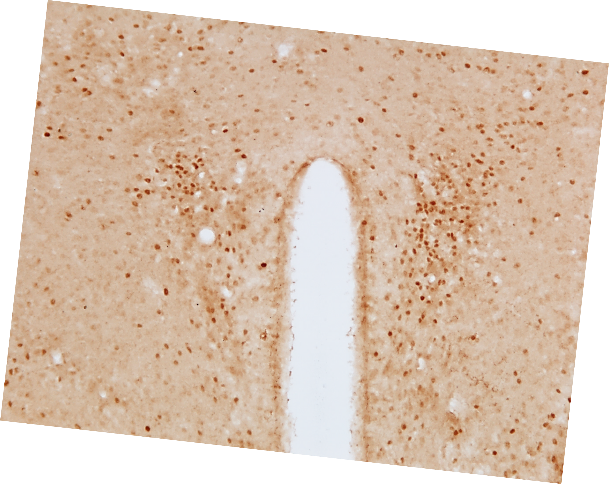

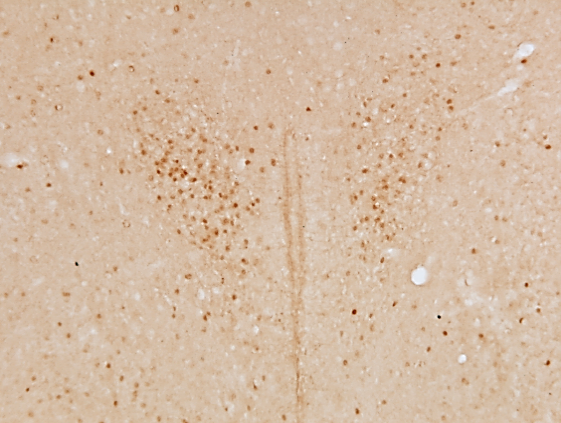


**Basal**

**FUST**

**TST**

**maLPA_1_-null**

**WT**

**100 µm**


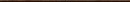

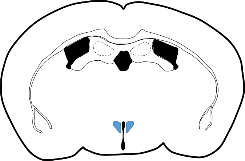

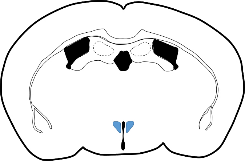

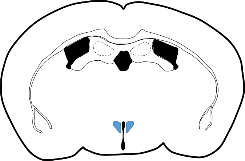


**a**

**PVN**

-0.70

Bregma -0.94


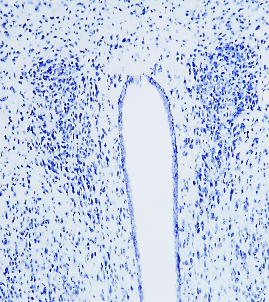


**PVN**

**V**


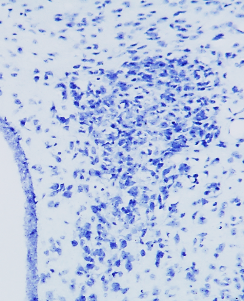


**PVN**

**V**

**b**

**c**

**d**

**e**

**f**

**g**

**h**

**i**

**j**

**Figure S3. Representative example of c-Fos positive immunoreactive nuclei in the paraventricular nucleus of the hypothalamus for each genotype in basal state and after behavioural test.** Bregma reference (**a**) and gallocyanin staining (**b**) are represented on the top as guide for PVN. MaLPA_1_-null mice displayed high c-Fos+ nuclei after TST as can be seen in (**i**) and (**j**) compared to wild-type mice in (**e**) and (**f**). (**c**)-(**e**) and (**g**)-(**i**) images were taken at x4 magnification; (**f**) and (**j**) images were taken at x40 magnification and are magnifications of (**e**) and (**i**) respectively. V: Ventricle.


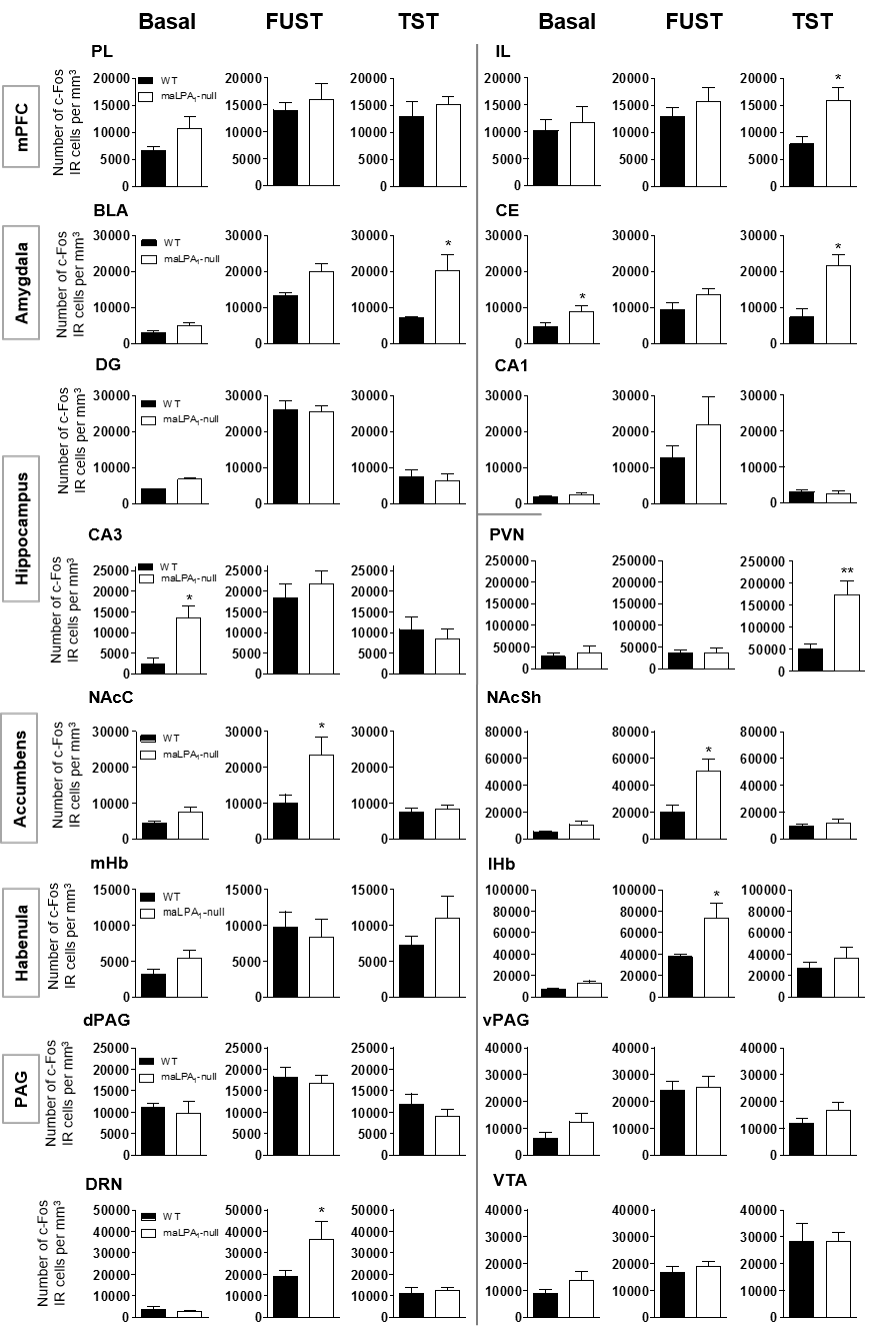


**a**


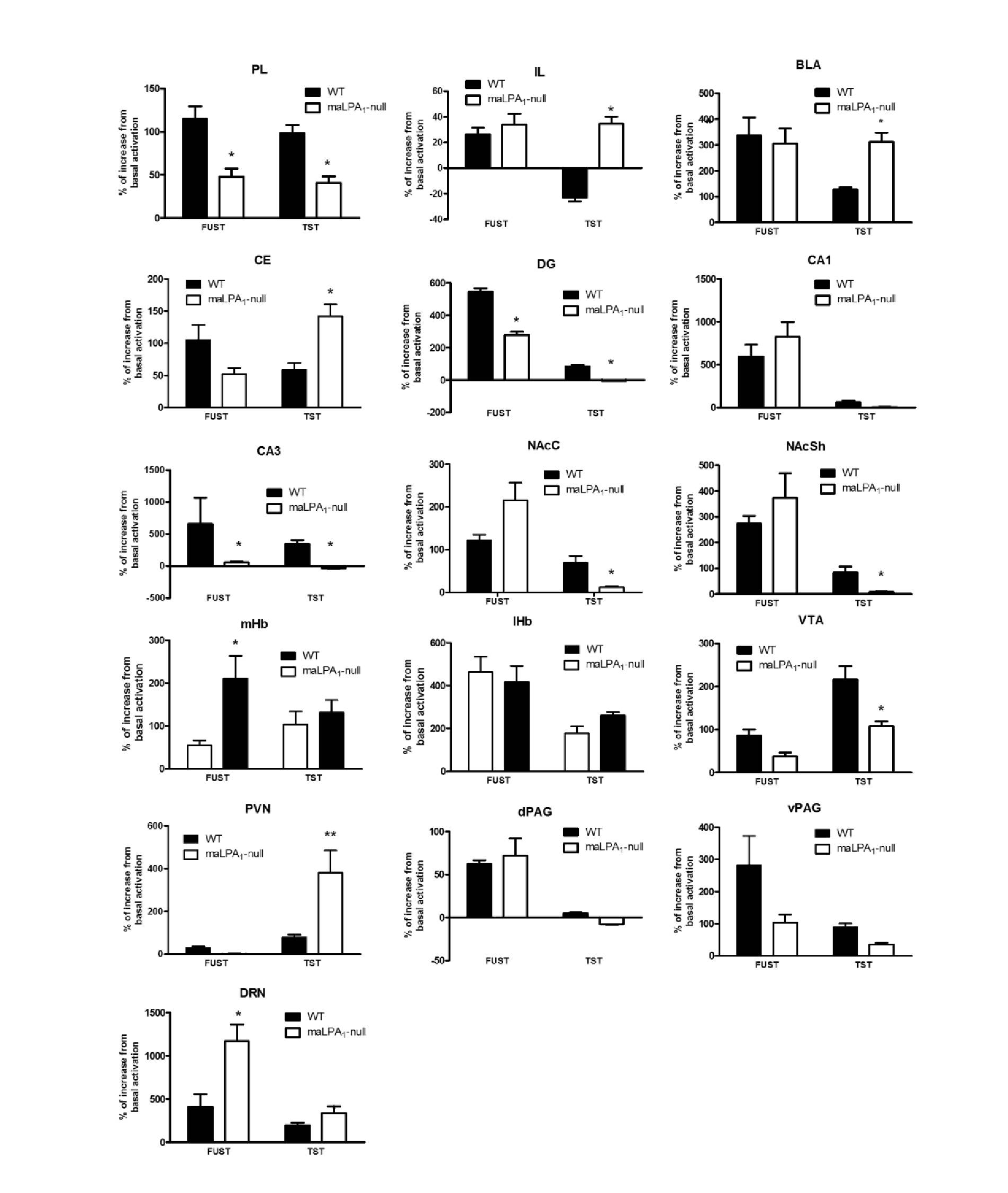


**b**

**c**

**c
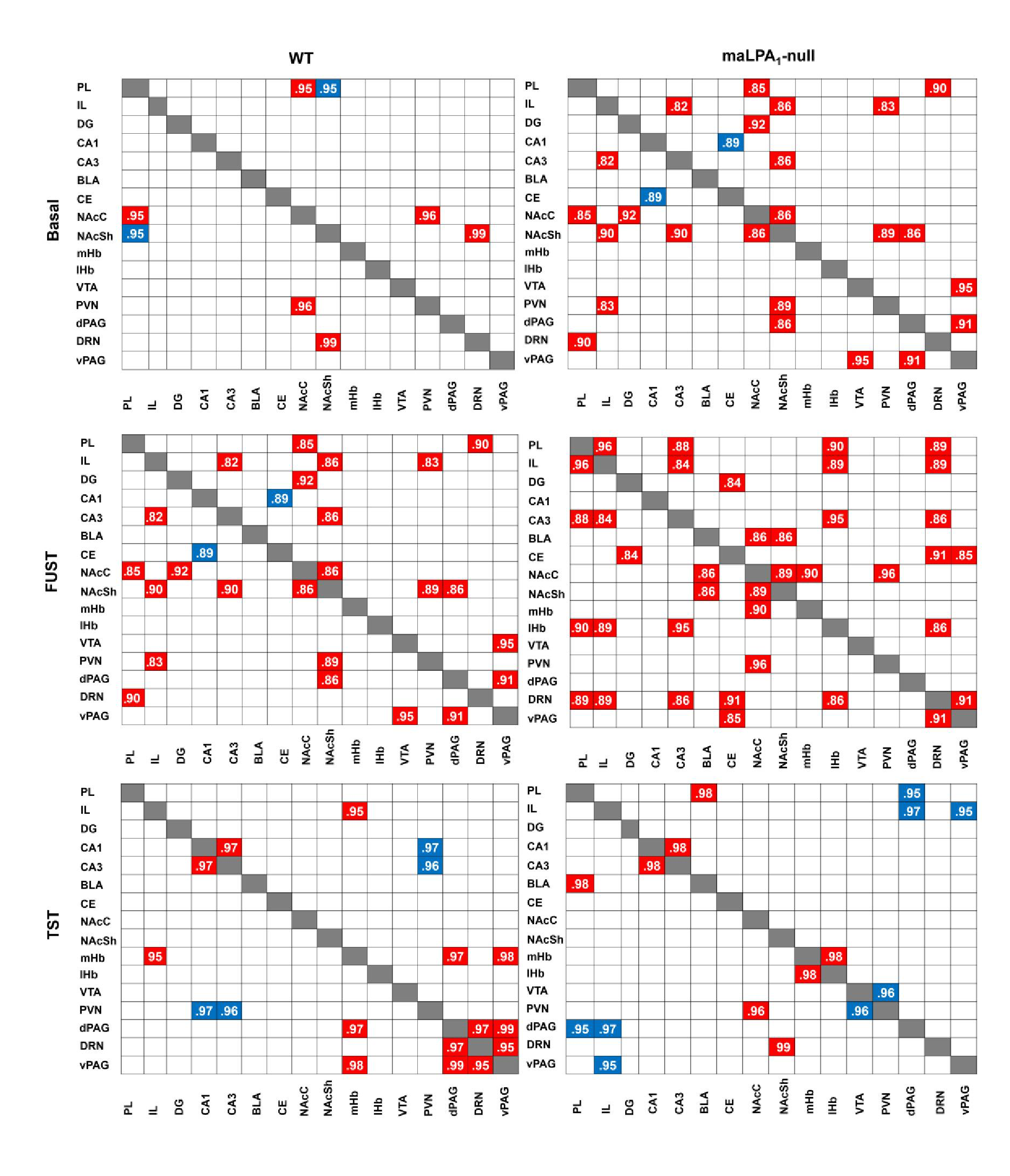
**

**d**
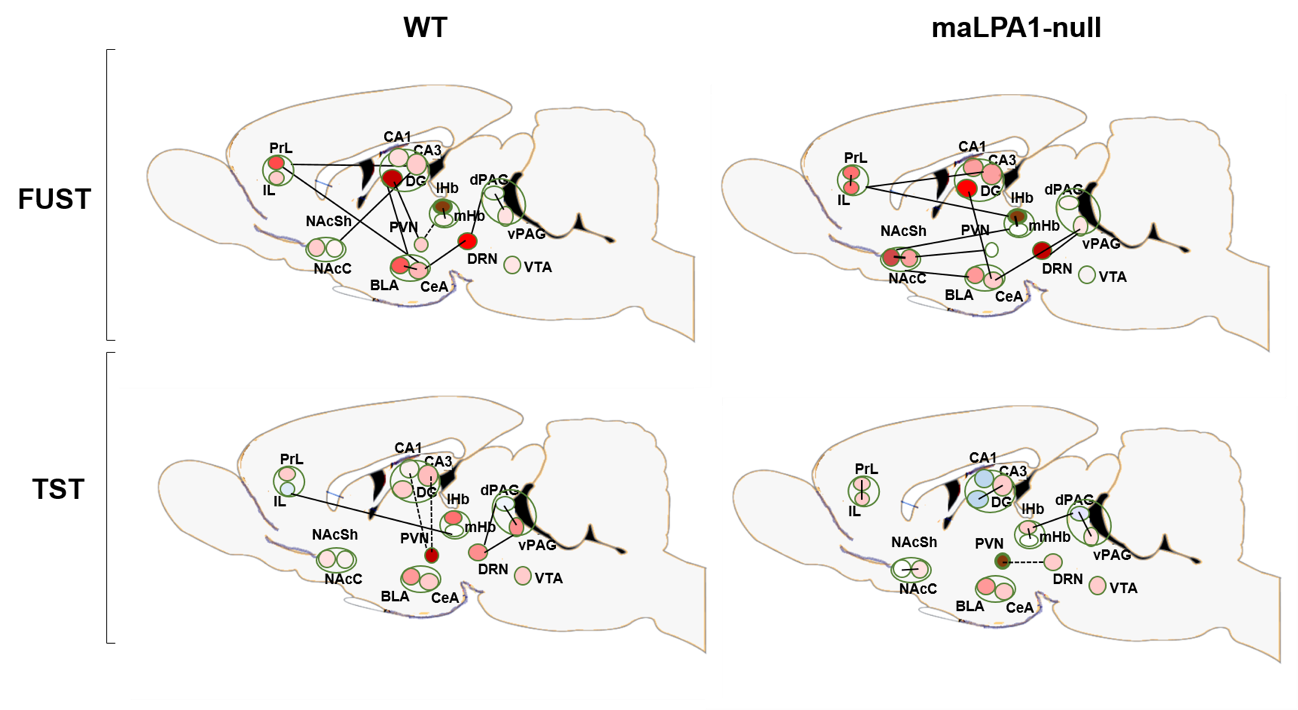


**Figure S4. Brain functional activation at baseline and after appetitive (FUST) or aversive (TST) stimuli. a** Abnormal activation of limbic and extralimbic structures in maLPA_1_-null mice after appetitive (FUST) or aversive (TST) stimuli. c-Fos expression is shown under the three conditions: basal, after FUST and after TST. Mice lacking the LPA_1_ receptor displayed significantly higher basal activity in CA3 and CE than wt mice, which might be related to a tendency to react in a maladaptive manner under stressful conditions and has been linked to the development of depression and anxiety disorder. In addition, maLPA_1_-null mice exhibited significantly higher activation of NAc, lHb and DRN after FUST and of IL, amygdala and PVN after TST; these regions are involved in processing negative stimuli and in emotional regulation, respectively, and play essential roles in the pathophysiology of depression. **b** The rate of change after completing FUST or TST indicates how the functional brain activation changes after completing the behavioural test in relation to baseline in the two genotypes. **c** Significant correlation between brain areas. Positive significant correlations are displayed in red squares with *r* values, and negative correlations are shown in blue. **d** Correlation of c-Fos expression between the assessed brain areas for each experimental procedure. Significant correlation between two areas is represented as a dotted (P < 0.05) or a continuous (P < 0.005) line. Pearson’s r ranged from 0.796 to 0.986 and was always positive except for the circled point. Importantly, the graphs represent functional connectivity but not a brain circuit because functionally linked areas may not be connected anatomically. * P<0.05 or P<0.01 with respect to wt animals. BLA, basolateral amygdala; CA1, cornus ammonis 1; CA3, cornus ammonis 3; CE, central amygdala; d/vPAG, dorsal/ventral periaqueductal grey matter; DG, dentate gyrus; DRN, dorsal raphe nucleus; FUST, Female Urine Sniffing Test; IL, infralimbic cortex; IR, immunoreactive; lHb, lateral habenula; mHb, medial habenula; mPFC, medial prefrontal cortex; NAc, nucleus accumbens; NAcC, nucleus accumbens core; NAcSh, nucleus accumbens shell; PL, prelimbic cortex; PVN, paraventricular nucleus; TST, Tail Suspension Test; VTA, ventral tegmental area.


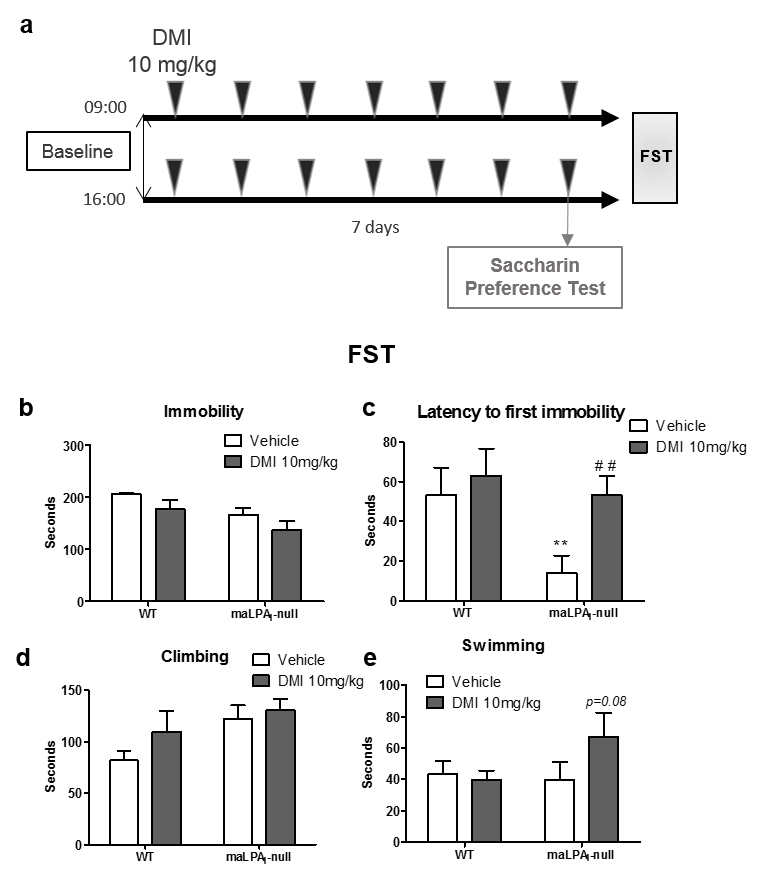


**Figure S5. Chronic desipramine administration decreases despair behaviour in maLPA_1_-null mice.**

**a** Experimental procedure in which animals received two daily administrations of desipramine or vehicle. **b-e** Desipramine treatment increased the latency to the first immobility period (c) and increased the adaptive active behaviour in the FST (e). **p< 0.01 with respect to wt animals. ##p<0.01 with respect to vehicle treatment.

**
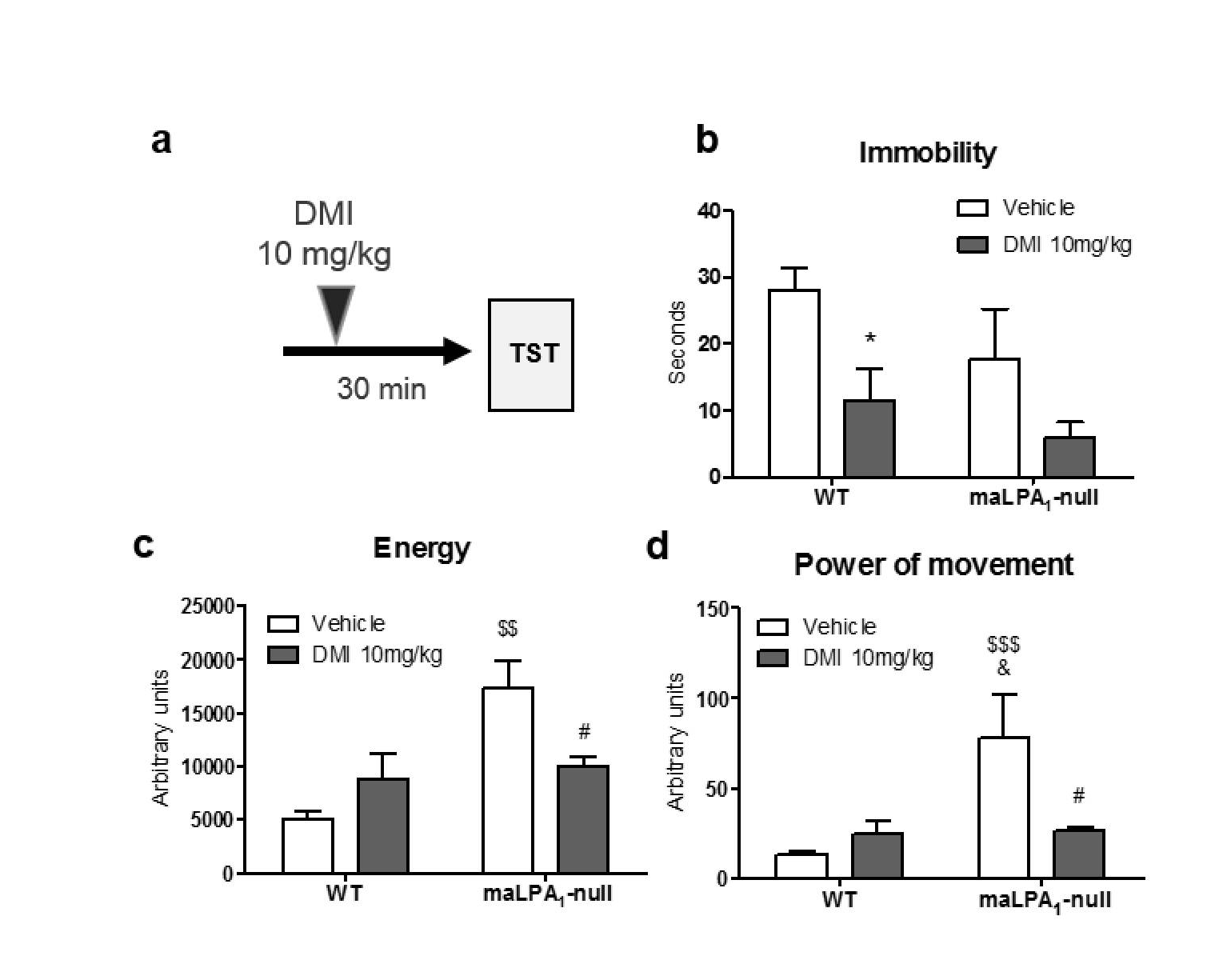
**

F(1,11)=8.08; P=0.016

Treatment x Genotype

F(1,11)=7.51; p=0.019

Treatment x Genotype

**Figure S6. Desipramine administration attenuates agitation in maLPA1-null mice.** Agitation as a symptom of depression has been significantly attenuated after i.p. desipramine administration in mice lacking the LPA_1_ receptor. *P<0.05 DMI-treated wt mice compared with vehicle-treated wt mice; # P<0.05 DMI-treated maLPA_1_-null mice compared with vehicle-treated null mice. $$ P<0.01; $$$ P<0.005 vehicle-treated null mice versus vehicle-treated wt mice. & P<0.05 vehicle treated wt mice compared with DMI-treated null mice. DMI = Desipramine.


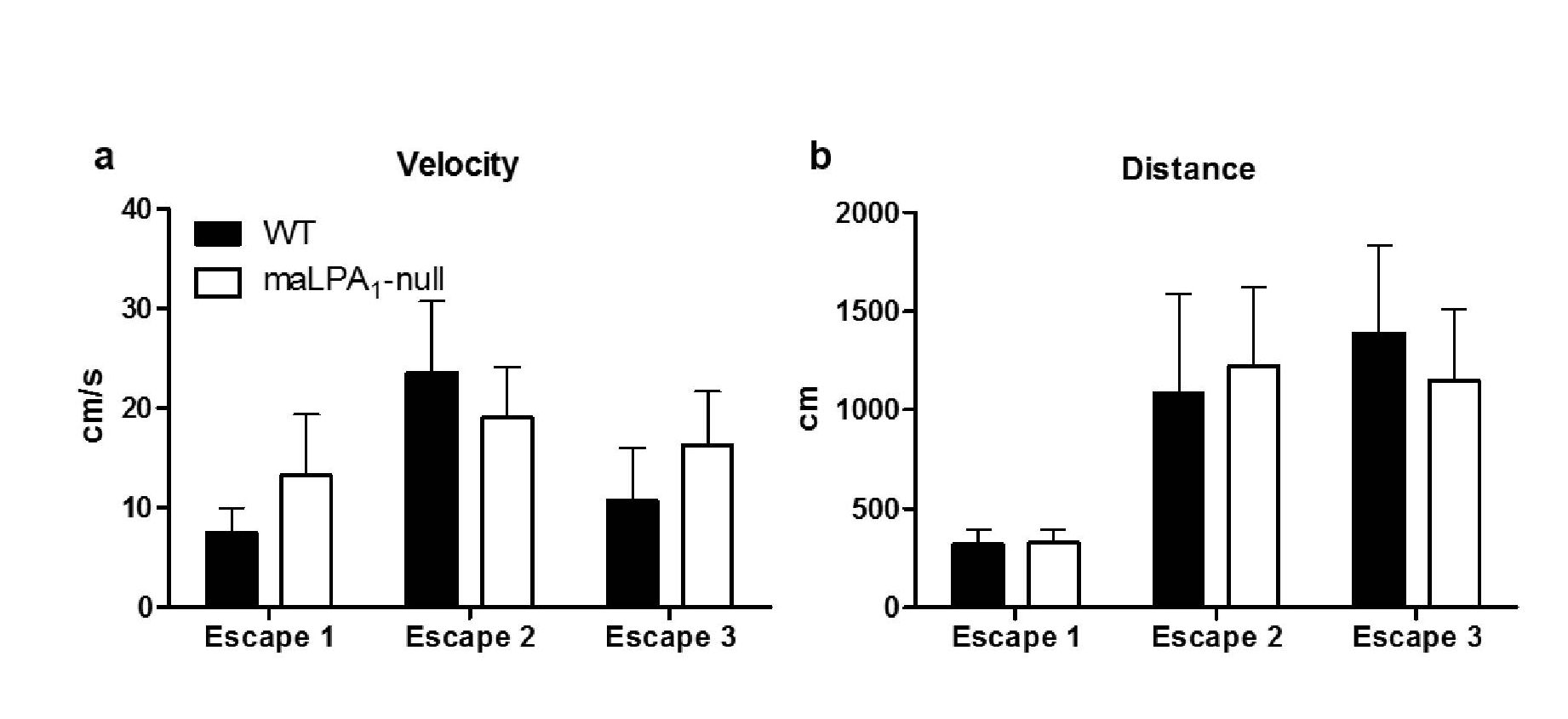


**Figure S7. Locomotion analysis in the escape test in the ETM.** No significant differences were observed in the overall velocity (cm/s) (**a**) or distance travelled through the open arms (**b**) in the T-maze. Data were analysed using Ethovision XT (Noldus, Wageningen, The Netherlands).

**
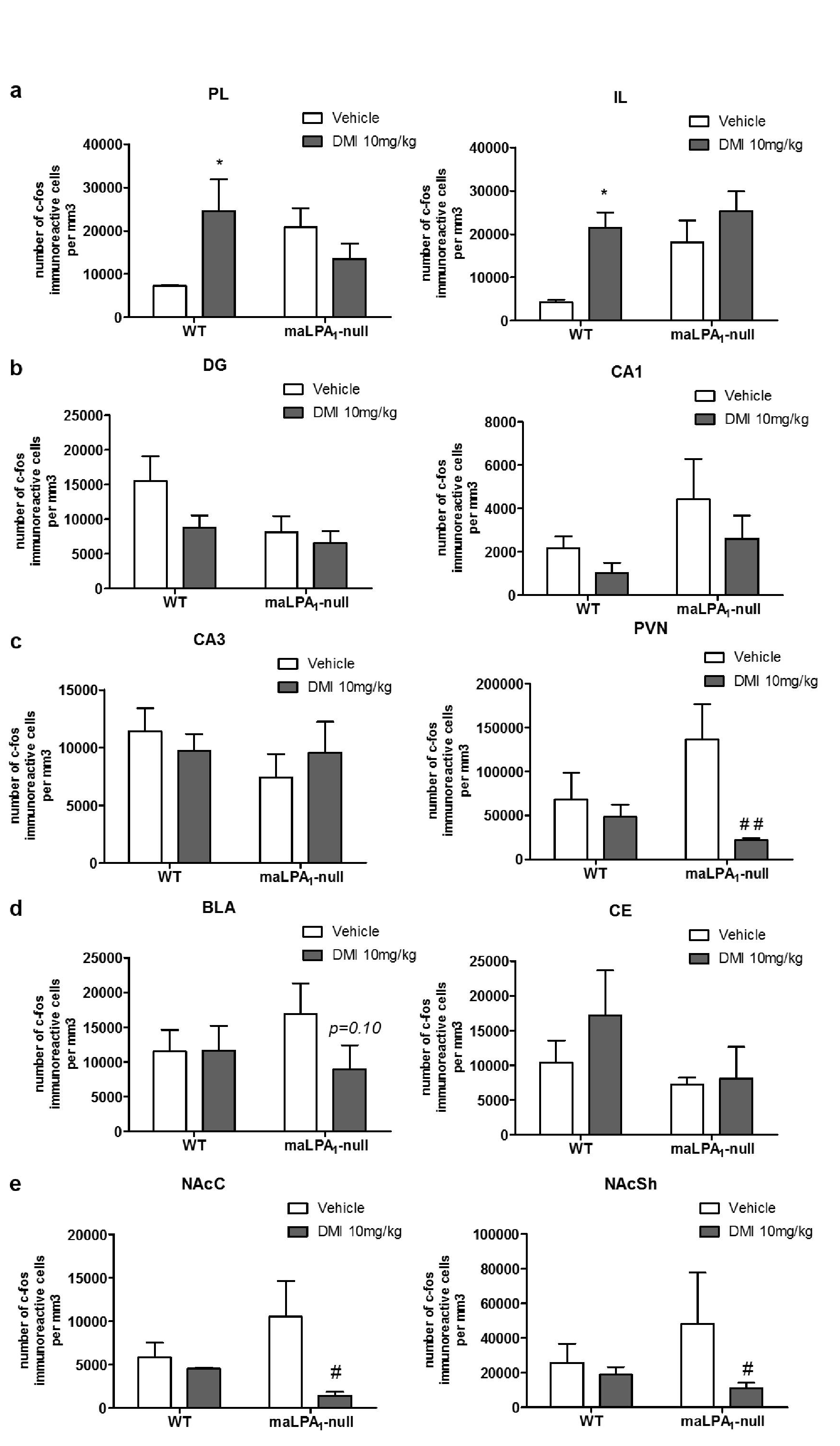
**

**f**

**
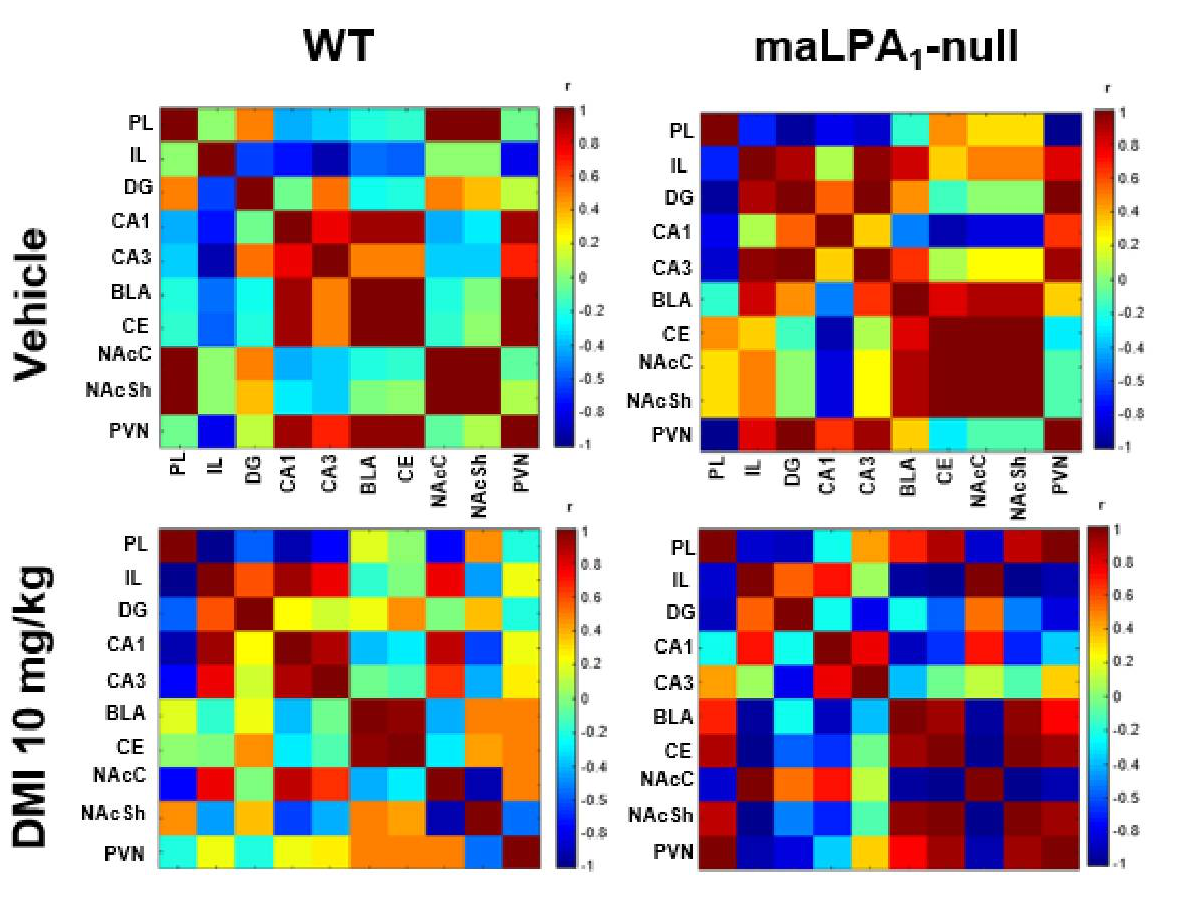
**

**Figure S8. Desipramine treatment reverted the anomalous brain activation observed in null mice after the TST. a-e** Desipramine treatment reduced the activity observed in the PVN and BLA, in which increased activity has been associated with dysfunctional ability to regulate emotion and negative mood bias, resulting in long-term depressive and anxious symptoms that have been related with anomalous stress coping and emotional regulation. **f** The treatment with DMI modified the interregional correlation matrices after the TST. * p<0.05 Differences with respect to vehicle-treated wt mice; # p<0.05 Differences with respect to vehicle-treated maLPA1-null mice. BLA, basolateral amygdala; CA1, cornus ammonis 1; CA3, cornus ammonis 3; CE, central amygdala; DG, dentate gyrus; IL, infralimbic cortex; NAcC, nucleus accumbens core; NAcSh, nucleus accumbens shell; PL, prelimbic cortex; PVN, paraventricular nucleus; TST, Tail Suspension Test.

**4.- Supplementary References**

1. Dávila G, Pedraza C, López-Ávalos M, Fernández-Llebrez P. Memoria olfativa de ratones machos adultos en ausencia del receptor LPA1 del ácido lisofosfatídico. In Gimenez A (ed). *Comportamiento y Palabra Estudios*. Universidad de Málaga, Spain, 2005, pp 15-16.

2. Malkesman O, Scattoni ML, Paredes D, Tragon T, Pearson B, Shaltiel G et al. The female urine sniffing test: a novel approach for assessing reward-seeking behavior in rodents. *Biol Psychiatry* 2010; **67**: 864-871.

3. Castilla-Ortega E, Pavón FJ, Sánchez-Marín L, Estivill-Torrús G, Pedraza C, Blanco E et al. Both genetic deletion and pharmacological blockade of lysophosphatidic acid LPA1 receptor results in increased alcohol consumption. *Neuropharmacology* 2016; **103**: 92-103.

4. Porsolt RD, Le Pichon M, Jalfre M. Depression: a new animal model sensitive to antidepressant treatments. *Nature* 1997; **266**: 730-732.

5. Cryan JF, Markou A, Lucki I. Assessing antidepressant activity in rodents: recent developments and future needs. *Trends Pharmacol Sci* 2002; **23**: 238-245.

6. Jacobson LH, Cryan JF. Feeling strained? Influence of genetic background on depression-related behavior in mice: a review. *Behav Genet* 2007; **37**: 171-213.

7. Cryan JF, Mombereau C, Vassout A. The tail suspension test as a model for assessing antidepressant activity: review of pharmacological and genetic studies in mice. *Neurosci Biobehav Rev* 2005; **29**: 571-625.

8. Khemissi W, Farooq RK, Le Guisquet AM, Sakly M, Belzung C. Dysregulation of the hypothalamus-pituitary-adrenal axis predicts some aspects of the behavioral response to chronic fluoxetine: association with hippocampal cell proliferation. *Front Behav Neurosci* 2014; **8**: 340.

9. Ritov G, Boltyansky B, Richter-Levin G. A novel approach to PTSD modeling in rats reveals alternating patterns of limbic activity in different types of stress reaction. *Mol Psychiatry* 2015; **21**: 630-641.

10. Castilla-Ortega E, Rosell-Valle C, Blanco E, Pedraza C, Chun J, Rodríguez de Fonseca F et al. Reduced wheel running and blunted effects of voluntary exercise in LPA1-null mice: the importance of assessing the amount of running in transgenic mice studies. *Neurosci Res* 2013; **7**: 170-179.

11. Dusaulcy R, Daviaud D, Pradère JP, Grès S, Valet P, Saulnier-Blache JS. Altered food consumption in mice lacking lysophosphatidic acid receptor-1. *J Physiol Biochem* 2009; **65**: 345-350.

12. Castilla-Ortega E, Sánchez-López J, Hoyo-Becerra C, Matas-Rico E, Zambrana-Infantes E, Chun J et al. Exploratory, anxiety and spatial memory impairments are dissociated in mice lacking the LPA1 receptor. *Neurobiol Learn Mem* 2010; **94**: 73-82.

13. Santin LJ, Bilbao a, Pedraza C, Matas-Rico E, López-Barroso D, Castilla-Ortega E et al. Behavioral phenotype of maLPA1-null mice: increased anxiety-like behavior and spatial memory deficits. *Genes Brain Behav* 2009; **8**:772-784.

14. Castilla-Ortega E, Pedraza C, Chun J, de Fonseca FR, Estivill-Torrús G, Santín LJ. Hippocampal c-Fos activation in normal and LPA₁-null mice after two object recognition tasks with different memory demands. *Behav Brain Res* 2012; **232**: 400-405.

15. Harrison S. LPA1 receptor-deficient mice have phenotypic changes observed in psychiatric disease. *Mol Cell Neurosci* 2003; **24**: 1170-1179.

16. Pedraza C, Sánchez-López J, Castilla-Ortega E, Rosell-Valle C, Zambrana-Infantes E, García-Fernández M, et al. Fear extinction and acute stress reactivity reveal a role of LPA(1) receptor in regulating emotional-like behaviors. *Brain Struct Funct* 2014; **219**: 1659-1572.

17. Castilla-Ortega E, Hoyo-Becerra C, Pedraza C, Chun J, Rodríguez De Fonseca F, Estivill-Torrús G, et al. Aggravation of chronic stress effects on hippocampal neurogenesis and spatial memory in LPA₁ receptor knockout mice. *PLoS One* 2011; 6: e25522.

18. Matas-Rico E, García-Diaz B, Llebrez-Zayas P, López-Barroso D, Santín L, Pedraza C, et al. Deletion of lysophosphatidic acid receptor LPA1 reduces neurogenesis in the mouse dentate gyrus. *Mol Cell Neurosci* 2008; **39**: 342-355.

19. Cunningham MO, Hunt J, Middleton S, LeBeau FEN, Gillies MJ, Gillies MG et al. Region-specific reduction in entorhinal gamma oscillations and parvalbumin-immunoreactive neurons in animal models of psychiatric illness. *J Neurosci* 2006; **26**: 2767-2776.

20. Roberts C, Winter P, Shilliam CS, Hughes ZA, Langmead C, Maycox PR et al. Neurochemical changes in LPA1 receptor deficient mice-a putative model of schizophrenia. *Neurochem Res* 2005; **30**: 371-377.

21. Musazzi L, Di Daniel E, Maycox P, Racagni G, Popoli M. Abnormalities in α/β-CaMKII and related mechanisms suggest synaptic dysfunction in hippocampus of LPA1 receptor knockout mice. *Int J Neuropsychopharmacol* 2011; **14**: 941-953.

22. Blanco E, Bilbao A, Luque-Rojas MJ, Palomino A, Bermúdez-Silva FJ, Suárez J, et al. Attenuation of cocaine-induced conditioned locomotion is associated with altered expression of hippocampal glutamate receptors in mice lacking LPA1 receptors. *Psychopharmacology* (Berl) 2012; **220**: 27-42.

23. García-Fernández M, Castilla-Ortega E, Pedraza C, Blanco E, Hurtado-Guerrero I, Barbancho MA, et al. Chronic immobilization in the malpar1 knockout mice increases oxidative stress in the hippocampus. *Int J Neurosci* 2012; **122**: 583-589.

24. García-Díaz B, Riquelme R, Varela-Nieto I, Jiménez AJ, de Diego I, Gómez-Conde AL et al. Loss of lysophosphatidic acid receptor LPA1 alters oligodendrocyte differentiation and myelination in the mouse cerebral cortex. *Brain Struct Funct* 2015; **220**: 3701-3720.
