# Supplementary material for: maLPA1-null mice as an endophenotype of anxious depression
Source: Transl Psychiatry. 2017 Apr 4;7(4):e1077–. doi: 10.1038/tp.2017.24 (PMC5416683; doi:10.1038/tp.2017.24)
Supplement: Supplementary Table S1 [file tp201724x2.doc]

| **Table S1.** maLPA_1_-null mice satisfied main validity criteria to be considered as an animal model of anxious-depression. | | |
| --- | --- | --- |
| **Face validity**  ***Behavioural manifestations are resemblance to symptoms observed in patients with anxious-depression.***  **Depression**  Loss of interest or pleasure (Anhedonia)  ***- Sweet solution preference***  ***- Female urine sniffing test***  *-* Reduced wheel running^10^*  Change in weight or appetite  - Altered food consumption^11^  Agitation. Emotional reactivity  ***- Forced swim test*****  ***- Tail suspension test***  Loss of energy or fatigue  ***- Nesting impairments***  - Reduced wheel running^10^*.  Cognitive impairment  - Working memory^12^  - Spatial memory^12,13^  - Episodic Memory (What-when-where task^14^)  - Prepulse inhibition^15^  **Anxiety**  - Open field^13^  - Elevated plus maze^13^  ***- Elevated T-maze***  - Hole-board test^14^  Fear  - Deficit in fear extinction^16^ | **DSM-5 criteria for major depressive disorder**  ***Five or more out of nine symptoms***  (including at least one of depressed mood and loss of interest or pleasure).  Depressed mood*  Loss of interest or pleasure (Anhedonia)  Change in weight or appetite  Insomnia or hypersomnia  Psychomotor retardation or agitation  Loss of energy or fatigue  Worthlessness or guilt*  Impaired concentration or indecisiveness  Thoughts of death or suicidal ideation or attempt*  **WITH ANXIOUS DISTRESS SPECIFIER CRITERIA**  ***Two or more out of five symptoms on most days of episode.***  Feeling keyed up or tense  Feeling unusually restless  Difficulty concentrating because of worry  Fear that something awful may happen  Feeling that the individual might lose control of herself*  _______________________  **Cannot be modeled in mice* | **Construct validity**  ***The pathophysiological changes that occur in patients with the disorder also occur in LPA_1_-null mice***  Changes in HPA-axis.  - Hypercortisolemia after acute stress^16^  - Hypocortisolemia after chronic stress^18^  Neuroanatomical changes  - Hippocampal atrophy^17,18^  - Reduce amygdala volume^16^  Neurofunctional changes  - Amygdala hyperreactivity^16^  ***- Impaired functional connectivity pattern***  Neurotransmission dysfunction  - GABA^16,19,20^  - Glutamate^20,21,22^  - Serotonin^15,20^  - Impairment of neurogenesis^17,18^  - Mitochondrial affectation^23^  - Increased oxidative stress^23^  - Glial alteration^24^  **Predictive validity**  ***Behavioural changes should be reversed by effective treatment.***  *- Desipramine improved anhedonic behaviour assessed by sweet solution preference (Figure 1).*  *- Desipramine increase latency to the first immobility period in FST i.e. increase motivation to escape of aversive situation (see Figure S5)*  *- Desipramine attenuates agitation as a symptom of depression in null mice in TST (Figure S6).*  *- Desipramine improves the altered nesting behavior as a symptom of fatigue (Figure 2).* |

In the middle, DSM-5 criteria for depression with anxious distress. On the left and right validity criteria that maLPA_1_-null mice meet to be considered as a good animal models of anxious-depression. Data are based in accumulated results and experimental results presented here (in black-bold letters). *Reduced wheel running could be conceptualised as sign of fatigue or a loss of motivation. ** Null mice exhibited reduced latency to first immobility period interpreted as an indicator of reduced intrinsic motivation to escape. *** In addition to this symptoms, null mice showed an incremented intake and preference for alcohol^75^. Both anxiety and depression frequently drive to alcohol abuse.
